# Synthetic Elaboration, DFT Profiling, and Molecular-Dynamics-Guided Computational Validation Toward Anti-Diabetic Therapeutics: Tailored Pyrimidine-Derived Pyrazole-Thiadiazole Hybrid Scaffolds

**DOI:** 10.3390/ph19060915

**Published:** 2026-06-10

**Authors:** Nahed Sail Alharthi

**Affiliations:** Department of Medical Laboratory, College of Applied Medical Sciences, Prince Sattam Bin Abdulaziz University, Alkharj 11942, Saudi Arabia; n-alharthi@hotmail.com

**Keywords:** pyrimidine, alpha-amylase, alpha-glucosidase, MD simulations, DFT, ADME analysis

## Abstract

**Background/Objectives**: Diabetes mellitus (DM) is a critical metabolic condition with escalated blood glucose levels caused by insulin resistance, restricted insulin production, and the activity of alpha-amylase and alpha-glucosidase enzymes. **Methods**: This current work focuses on the synthesis and evaluation of novel Pyrimidine-derived pyrazole-based thiadiazole derivatives to target DM by inhibiting α-amylase and α-glucosidase. **Results**: The findings exhibited that, except for three compounds, all other synthesized derivatives inhibited α-amylase and α-glucosidase enzymes with *IC*_50_ values ranging from 5.17 μM to 29.84 μM on α-amylase and 7.60 μM to 31.62 μM on α-glucosidase, in comparison to the standard drug Acarbose (α-amylase *IC*_50_ = 8.25 ± 0.80 μM; α-glucosidase *IC*_50_ = 10.75 ± 1.10 μM). Analogs **8g**, **8k**, and **8b** displayed superior or comparable inhibitory activity compared to the reference drug Acarbose. The inhibition potential of the derivatives can be attributed to their stable contacts with crucial amino acid residues of targeted enzymes, as shown through molecular docking analysis. Moreover, DFT-calculated HOMO–LUMO parameters and electrostatic potential (ESP) maps were used to gain complementary insight into the electronic characteristics, charge distribution, and potential interaction behavior of the synthesized derivatives, which supported the molecular docking observations. **Conclusions**: Experimental outcomes and in silico support display that these derivatives serve as potential leads for anti-diabetic drug development. These potent pyrimidine-derived pyrazole-based thiadiazole derivatives were comparable to an existing diabetic mellitus inhibitor, specifying potential for further therapeutic development and optimization against diabetic mellitus.

## 1. Introduction

Diabetes mellitus (DM) is a long-term metabolic disease characterized by sustained hyperglycemia due to impairments in insulin release, insulin action, or both [1]. It is one of the greatest health problems in the world, especially in the developing world, where a high rate of urbanization, sedentary living, and change in diet have led to the rising prevalence of the condition [2]. As per the latest estimates, the count of diabetic patients is set to explode in the forthcoming decades, and it will be a significant strain on healthcare systems across the globe. Diabetes results in chronic hyperglycemia that causes serious complications such as cardiovascular diseases, neuropathy, nephropathy, and retinopathy, which eventually lead to a poor quality of life and higher mortality [3]. Diabetes management focuses mainly on blood glucose control, especially after meals. The use of enzymes that break down carbohydrates, such as a-amylase and a-glucosidase, has proven effective as a therapeutic strategy [4]. These enzymes are important in breaking down complex carbohydrates into glucose, and their inhibition slows glucose absorption and, hence, postprandial glucose spikes. Inhibitors like acarbose, used clinically, have proven effective but also have some undesirable gastrointestinal side effects, including bloating, diarrhea, and abdominal discomfort, which limit patient compliance and necessitate the development of more effective and safer alternatives [5]. Diabetes mellitus, particularly type 2 diabetes mellitus (T2DM), is a complex metabolic disorder associated with impaired glucose metabolism, inflammation, intestinal barrier dysfunction, lipid metabolism abnormalities and diabetic complications, where gut microbiota modulation, immune regulation, and therapeutic targeting of biomarkers such as ICAM1 and protein tyrosine phosphatase 1B have emerged as promising strategies for disease management and diagnosis [6,7,8,9,10,11]. Over the last few years, medicinal chemistry has drawn much attention to the design and development of heterocyclic compounds owing to their exceptional biological properties and structural versatility [12]. Nitrogen-containing heterocyclic compounds have attracted considerable attention in medicinal and synthetic chemistry due to their diverse biological activities and broad pharmaceutical applications, including antimicrobial, anticancer, and enzyme inhibitory properties, making them valuable scaffolds for the development of novel therapeutic agents [13,14,15,16]. Heterocycles based on nitrogen, oxygen, and sulfur are common in bioactive molecules, pharmaceuticals, and natural products, and they are crucial for regulating biological targets [17]. Of these, triazoles, oxadiazoles, pyridines, and thiadiazoles have attracted considerable interest due to their pharmacological functions, including antimicrobial, anticancer, anti-inflammatory, and antidiabetic effects [18,19]. The scaffolds have high binding affinity for biological macromolecules via hydrogen bonds, π-π stacking, and electrostatic interactions, making them the best candidates for inhibiting enzymes. Molecular hybridization is a concept that has become one of the potent techniques in contemporary drug design [20]. It is a technique that entails joining two or more pharmacologically active moieties into a single framework molecule to promote biological activity, selectivity, and pharmacokinetics. Hybrid molecules tend to have synergies and enhanced efficacy compared to their constituent parts. Hybrid heterocyclic systems that combine triazole and oxadiazole structures have shown promising results as dual inhibitors of *α*-amylase and *α*-glucosidase in antidiabetic drug discovery [21]. These hybrids have a higher affinity for enzyme active sites due to strong interactions between electron-rich heteroatoms and aromatic systems, thereby increasing their inhibitory activity. Triazole derivatives, especially 1,2,3- and 1,2,4-triazoles, are known to be stable, bioavailable, and have a broad spectrum of biological activity [22]. The compounds can establish numerous non-covalent interactions with biological targets, thereby influencing their therapeutic potential. On the same note, 1,3,4-oxadiazole derivatives have attracted considerable interest due to their favorable pharmacokinetic properties and their ability to serve as bioisosteres to amides and esters. The compounds exhibit broad biological properties, including antidiabetic, anti-inflammatory, and enzyme-inhibitory effects [23]. Functionalities such as pyridine and thiol also enhance the biological profile of these molecules by increasing their binding affinity and electronic properties. Recent research has shown that heterocyclic hybrids with a triazole linkage exhibit strong inhibitory activity against *α*-amylase and *α*-glucosidase enzymes [24]. As an example, triazole-pyridine-oxadiazole hybrids were reported to exhibit strong enzyme inhibition, with molecular docking and density functional theory (DFT) studies indicating that they are effective antidiabetic agents [21]. Moreover, heterocyclic scaffolds are useful in drug discovery, with pyrimidine- and pyrazole-based heterocycles demonstrating antidiabetic activity [25]. The results also highlight the need to create new hybrid molecules that are more effective and less harmful.

Computational methods have played a major role in accelerating the identification and optimization of bioactive compounds, owing to their integration into drug discovery [26]. Molecular docking experiments provide information on the binding interactions between ligands and target enzymes, enabling the prediction of binding affinity and the mechanism of action [27]. Likewise, calculations in density functional theory (DFT) can provide useful information about the electronic properties of molecules, including HOMO-LUMO energies, energy gaps, and reactivity descriptors, which are directly related to the molecule’s chemical reactivity and biological activity [28]. Moreover, in silico ADME/ADMET tests are essential for determining the pharmacokinetic and toxicity characteristics of potential drug candidates, thereby minimizing the risk of failure at subsequent drug development stages [29].

Although significant progress has been made in the development of heterocyclic antidiabetic agents, there remains a need for new compounds with improved potency, selectivity, and safety profiles. Specifically, the progress of dual inhibitors of *α*-amylase and *α*-glucosidase is of special interest, since these substances can offer a better control of postprandial hyperglycemia. Furthermore, how experimental biological activity correlates with computational studies, such as docking, DFT, and ADMET analysis, is also a significant field of study towards rational drug design.

Here, the current research paper aims to design and synthesize new heterocyclic hybrids that incorporate a series of pharmacophores, thereby enhancing antidiabetic activity. The assessed compounds were tested for inhibition of *α*-amylase and *α*-glucosidase enzymes. Additionally, molecular docking experiments were performed to investigate binding interactions and the mechanism of inhibition, and DFT calculations were used to study the compounds’ electronic properties and reactivity. Moreover, in silico ADME and toxicity tests were performed to determine the drug-likeness and pharmacokinetic characteristics of the synthesized derivatives.

## 2. Results and Discussion

### 2.1. Chemical Synthesis

**Scheme 1:**
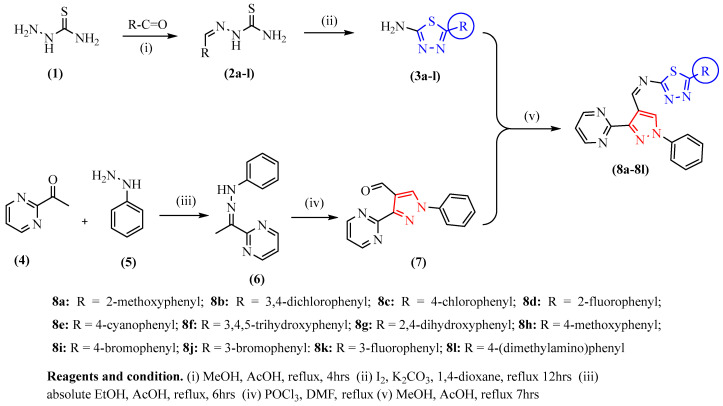
Synthesis of pyrazole-based thiadiazole derivatives (**8a**–**8l**). Red color indicate the pyrazole ring while blue color indicate the thiadiazole with substituted (R) group.

The pyrazole-based thiadiazole derivatives (**8a**–**8l**) were synthesized via a five-step sequence, with hydrazinecarbothioamide (**1**) as the starting reagent (Scheme 1). Initially, this moiety **1** was reacted with various substituted aldehydes in a methanol medium, which further went through iodine-mediated cyclization to yield 5-substituted-1, 3, 4-thiadiazol-2-amine (**3a**–**3l**). In a round-bottom flask, 1-(pyrimidin-2-yl)ethan-1-one (**4**) was reacted with phenylhydrazine (**5**) in ethanol medium utilizing acetic acid as a catalyst to afford the intermediate **6**. Then, the intermediate **7** was successfully synthesized by the introduction of the pyrazole-4-carbaldehyde unit on **6** using the Vilsmeier–Haack reaction. In the last step, **7** was reacted with different 5-substituted-1,3,4-thiadiazol-2-amine (**3a**–**3l**) in a methanol medium to afford the desired products (**8a**–**8l**). The Supplementary Information File contains full experimental details for each compound.

### 2.2. In Vitro α-Amylase and α-Glucosidase Inhibition

Diabetes mellitus is a critical metabolic condition with escalated blood glucose levels caused by insulin resistance, restricted insulin production, and the activity of α-amylase and α-glucosidase enzymes. In this work, synthetic pyrazole-based thiadiazole derivatives (**8a**–**8l**) target these enzymes in comparison to the standard drug Acarbose (α-amylase *IC*_50_ = 8.25 ± 0.80 μM; α-glucosidase *IC*_50_ = 10.75 ± 1.10 μM) [30]. Derivatives **8g**, **8k**, and **8b** displayed superior or comparable inhibitory activity on both enzymes compared to the reference drug used. The incorporation of versatile substituents on phenyl rings, including their position, nature, and number, affects the activity of the compound. Table 1 illustrates the attached substituents on the phenyl ring, along with their biological potential (*IC*_50_ ± SD in μM) on both α-amylase and α-glucosidase enzymes.

The SAR assessment underscores how each moiety and substituent influences the biological potential of the derivative against both enzymes. Analog **8g** (α-amylase *IC*_50_ = 5.17 ± 0.60 μM; α-glucosidase *IC*_50_ = 7.60 ± 0.80 μM) is the series’ lead compound, with two hydroxyl groups in ortho and para positions. These advantageous positions can activate the phenyl ring, leading to hydrogen bonding and other important interactions with the crucial residues of the catalytic sites in both enzymes, which is additionally supported by the docking results reviewed in Section 2.3. In comparison to that, compound **8f** (α-amylase *IC*_50_ = 16.40 ± 1.60 μM; α-glucosidase *IC*_50_ = 18.78 ± 1.70 μM), bearing three hydroxyl groups at the meta and para positions, resulted in a 3.2-fold and 2.5-fold reduced inhibitory activity against α-amylase and α-glucosidase activity. This can be attributed to the unfavorable substitution of the –OH group at the meta position, which may cause optimal geometry disruption and reduction in effective interactions due to steric/electronic crowding.

The inhibitory activity of compound **8k** (α-amylase *IC*_50_ = 6.83 ± 0.70 μM; α-glucosidase *IC*_50_ = 8.42 ± 0.90 μM) is also slightly better than the reference drug, due to its attached 3-fluorophenyl substituent. This specifies that the incorporation of a small electron-withdrawing group at the third position is favorable for binding and interactions with key amino acid residues. As in α-glucosidase, it was involved in the interaction with GLN182, ASP69, and TYR72. In the case of α-amylase, it showed only weak polar interactions. However, the replacement of this substituent with 2-fluorophenyl in compound **8d** revealed about 3.6-fold and 3.3-fold decreased inhibition potential against α-amylase (*IC*_50_ = 24.70 ± 1.80 μM) and α-glucosidase (*IC*_50_ = 27.39 ± 1.90 μM), respectively. The derivative **8b** (α-amylase *IC*_50_ = 8.13 ± 0.80 μM; α-glucosidase *IC*_50_ = 10.32 ± 0.90 μM) is the third most potent compound in the series. In this case, the attached substituent, 3, 4-dichlorophenyl, is favorable for binding, as it may establish hydrophobic interactions with the binding pocket of the enzymes. In both enzymes, the substituent interacted with the binding site through hydrophobic contacts. Compound **8e**, featuring a 4-cyanophenyl substituent, also displayed good inhibition potential (α-amylase *IC*_50_ = 12.64 ± 1.20 μM; α-glucosidase *IC*_50_ = 14.75 ± 1.30 μM), further supporting the role of the electron-withdrawing group positively. That was additionally supported by the docking analysis, as it formed a hydrogen bond with GLN63 in the α-amylase binding site and with ARG213 in the case of α-glucosidase. Compound **8c**, having a chloro group at only the para position, resulted in the inhibitory activity of *IC*_50_ = 15.78 ± 1.30 μM against α-amylase and *IC*_50_ = 17.49 ± 1.50 μM against α-glucosidase.

Replacement with a 4-methoxyphenyl substituent in compound **8h** (α-amylase *IC*_50_ = 20.67 ± 1.80 μM; α-glucosidase *IC*_50_ = 22.13 ± 1.90 μM) and a 2-methoxyphenyl substituent in compound **8a** (α-amylase *IC*_50_ = 29.84 ± 2.10 μM; α-glucosidase *IC*_50_ = 31.62 ± 2.20 μM) revealed reduced activity, indicating that electron-donating groups are less favorable for α-amylase and α-glucosidase inhibition. Interestingly, both bromo-substituted compounds (**8i** and **8j**) and dimethylamino-substituted (**8l**) exhibited no activity against both enzymes, which could be due to steric bulk of these groups and unfavorable electronic effects. Collectively, the SAR trends revealed that electron-donating groups are less favorable for α-amylase and α-glucosidase inhibition, except for –OH groups in ortho and para positions which contributed to the inhibitory activity positively, but –OH group at the meta position caused reduction in activity. Similarly, substituents containing bulky groups exhibited no activity. In comparison, electron-withdrawing groups at the specific position of the substituent resulted in potent activity.

### 2.3. Docking Studies

Four potent compounds in an enzyme inhibition assay were docked against the enzymes α-amylase and α-glucosidase, using acarbose as a control drug to assess their binding energies and interactions. Proteins were retrieved from the protein databank (RCSB) using their codes.

**Target α-amylase** (PDB ID: 1B2Y)

- Resolution: 3.20 Å

This was selected for its well-defined catalytic residues and its well-known use in an inhibitor screening study, with acarbose as a reference. Its structural stability and clarity make it ideal for reproducible docking experiments.

- Co-crystallized ligand: Acarbose
- Organism(s): Homo sapiens
- Chain utilized: Chain A

**Target α-glucosidase** (PDB ID: 3A4A)

- Resolution: 1.60 Å

This PDB ID was chosen for its high-resolution crystal structure of α-glucosidase, which has a well-defined active site and co-crystallized inhibitor. This enables accurate identification of the catalytic pocket and successful validation of docking.

- Co-crystallized ligand: alpha-D-glucopyranose
- Organism(s): Saccharomyces cerevisiae
- Chain utilized: Chain A

Redocking the native ligand resulted in a 0.8332 Å RMSD between the experimental and docked poses for α-amylase and 0.3857 Å RMSD for α-glucosidase, signifying reliable binding mode reproduction (RMSD < 2.0 Å accepted) (Figure S1 in the Supplementary Information File).

Discovery Studio Visualizer was employed to show and examine poses with the highest binding affinity throughout the docking process. The study found that potent derivatives efficiently interact with various amino acids in specific enzymes, resulting in remarkable results. Docking outcomes are visualized as three-dimensional (3D) and two-dimensional (2D). Three-dimensional visualization displays ligand fitting in a receptor site or active pocket of the targeted enzyme complex, while 2D visualization represents derivatives’ interactions with varying binding distances and structural components. Table 2 illustrates the binding interactions between the compounds and the interacting residues.

The crucial hydrophilic amino acid residues in the binding site of α-amylase includes GLU233, ASP300, ASP197, GLN63, HIS101, and THR163, while hydrophobic residues include LEU162, ALA198, TRP59, TYR62, HIS201, and ILE235. The catalytic triad for substrate catalysis in α-glucosidase consists of ASP215, GLU277, and ASP352. In addition to the catalytic residues, ASP62, TYR72, and ARG442 are located near the active site and, hence, participate in the catalytic process. The binding site of α-glucosidase, consisting of TYR389, TYR158, HIS280, ASP69, TRP58, PHE301, PHE303, TYR347, TYR387, ARG446, ASP409, VAL410, and GLU408, mediates hydrophilic and hydrophobic interactions with the substrate and competitive inhibitor.

#### 2.3.1. Protein–Ligand Interaction Profile of Acarbose with the Active Site of α-Amylase and α-Glucosidase

**Figure 1:**
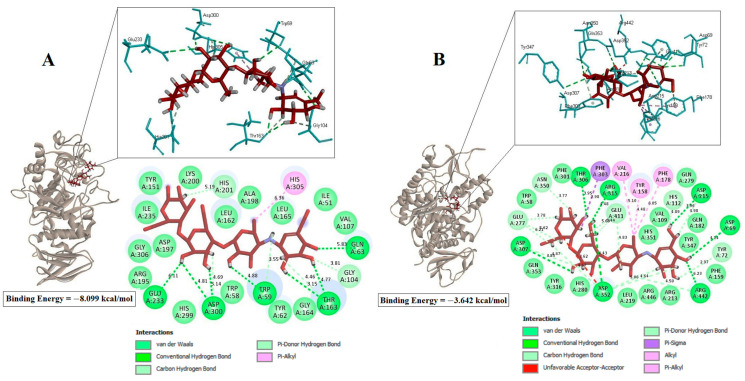
Interactivity profile of acarbose against (**A**) α-amylase and (**B**) α-glucosidase.

Acarbose has the highest binding affinity (−8.099 kcal/mol) compared with the tested compounds and significant interaction with α-amylase, making it extremely stable (Figure 1A). It formed seven conventional hydrogen bonds with important catalytic and neighboring residues of the active site, which include ASP300, THR163, TRP59, GLN63, and GLU233. Extensive van der Waals interactions and carbon-based hydrogen bonds resulted in efficient pocket filling and non-classical stabilization. Despite that, only one long-distance π-alkyl contact was noticed, and strong π-stacking interactions were missing, which may limit the hydrophobic stability within the binding pocket.

In the binding site of α-glucosidase, it showed the lowest binding affinity (−3.642 kcal/mol) compared with the binding affinities of the tested compounds, but its interaction profile is extensive and stable, as it includes seven conventional hydrogen bonds with crucial residues for strong polar anchorage (Figure 1B). Additionally, it formed various C-H contacts participating in ligand accommodation and van der Waals interactions. Similar to its interaction profile against α-amylase, strong π-stacking contacts were absent, as well as the presence of an unfavorable acceptor–acceptor contact signifying electrostatic repulsion, which may be the destabilizing factor for the protein–ligand complex.

#### 2.3.2. Protein–Ligand Interaction Profile of Potent Synthesized Pyrazole-Based Thiadiazole Derivatives with the Active Site of α-Amylase

**Figure 2:**
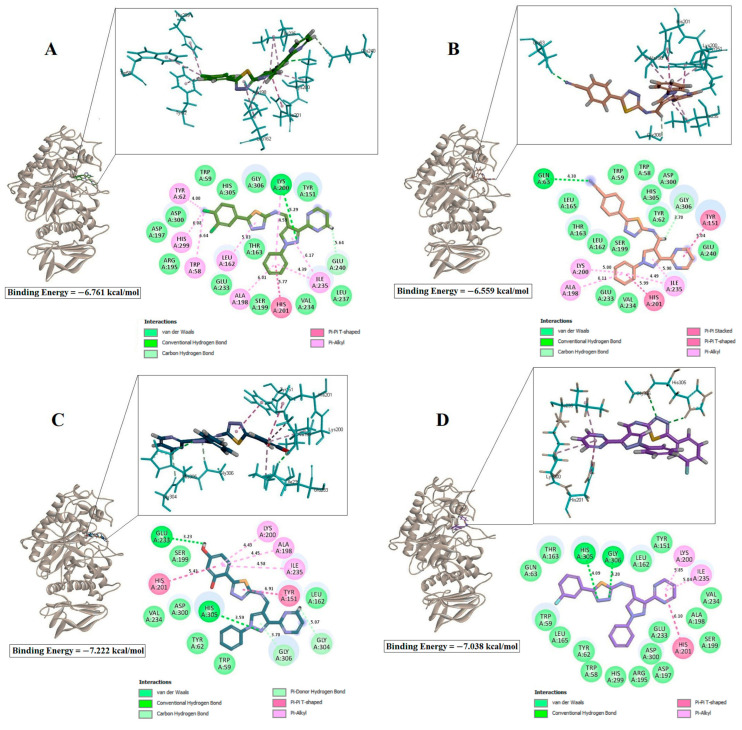
3D and 2D interactivity profile of (**A**) **8b**, (**B**) **8e**, (**C**) **8g**, and (**D**) **8k** against α-amylase.

Figure 2 displays how potent synthesized pyrazole-based thiadiazole derivatives bind (**8b**, **8g**, **8k**, and **8e**) to the α-amylase enzyme active site and relates to the proposed pharmacophoric model. The tested compounds are positioned deeply inside the catalytic site and have robust surface complementarity with the cavity of the enzyme, resulting in a stable ligand–protein complex. The ligand’s arrangement permits its main scaffolds and substituents to cross the active site, engaging numerous interaction areas simultaneously. The two-dimensional interaction map demonstrates that compounds establish critical conventional hydrogen bonds with catalytically significant residues; for instance, **8g** interacts with GLU233 and HIS305, **8k** with HIS305 and GLY306, **8b** with LYS200, and **8e** with GLN63, signifying the essential donor and acceptor pharmacophoric properties. The ligands **8g** and **8e** aromatic rings engage with aromatic amino acid residues HIS201 and TYR151, while **8k** and **8b** engage with HIS201, through π-π stacking and π-π T-shaped interactions. These contacts resulted in aromatic pharmacophoric elements that improve binding stability, a feature lacking in the standard drug. Numerous hydrophobic contacts, carbon-based hydrogen bonds, and extensive van der Waals interactions are also noticed, displaying good hydrophobic complementarity, non-classical stabilization, and pocket filling. Overall, compound **8g** exhibits high biological activity (*IC*_50_ = 5.17 ± 0.60 µM) against α-amylase and has a favorable docking score of -7.222 kcal/mol, comparable with the standard drug, representing stable binding within the enzyme active site and potential as an inhibitor. Additionally, compound **8k** (*IC*_50_ = 6.83 ± 0.70 µM) was ranked second, showing a docking score value of −7.038 kcal/mol, **8b** (*IC*_50_ = 8.13 ± 0.80 µM) as third (binding affinity = −6.761 kcal/mol), and **8e** (*IC*_50_ = 12.64 ± 1.20 µM) as fourth (binding affinity = −6.559 kcal/mol).

#### 2.3.3. Protein–Ligand Interaction Profile of Potent Synthesized Pyrazole-Based Thiadiazole Derivatives with the Active Site of α-Glucosidase

**Figure 3:**
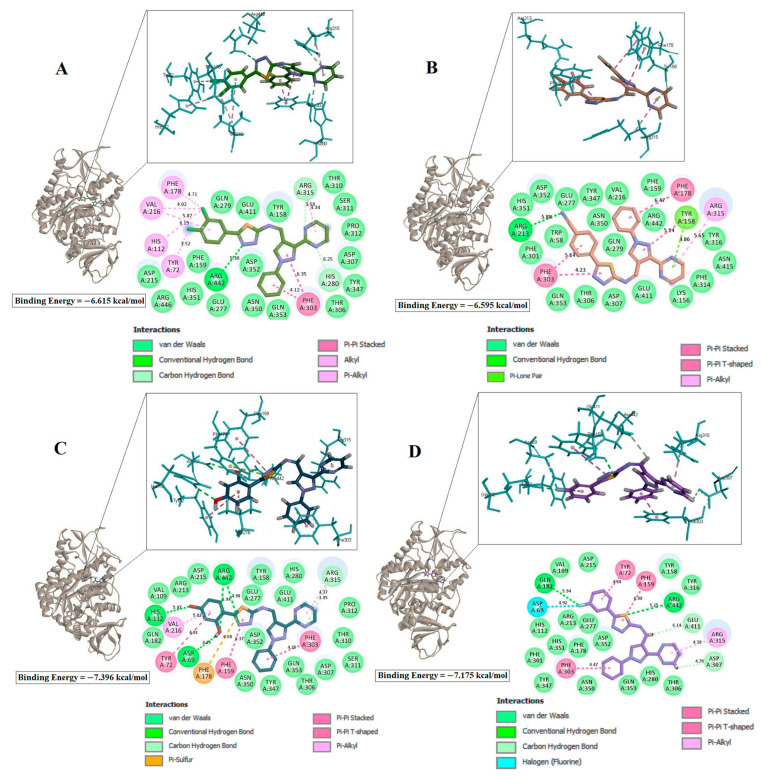
3D and 2D Interactivity profile of (**A**) **8b**, (**B**) **8e**, (**C**) **8g**, and (**D**) **8k** against α-glucosidase.

Figure 3 shows how **8b**, **8g**, **8e**, and **8k** bind to the α-glucosidase active site, favoring the hypothesized pharmacophoric model. These compounds are placed deeply inside the catalytic site and have strong surface complementarity with the cavity of the enzyme, resulting in a stable ligand–protein complex. The ligand’s alignment facilitates its main scaffold (pyrazole/thiadiazole) or its substituents to cross the active site, occupying numerous interaction areas simultaneously. The two-dimensional interaction map demonstrates that compounds establish critical conventional hydrogen bonds with catalytically significant residues, underscoring the acceptor and donor pharmacophoric properties. In compound **8g**, the thiadiazole ring’s N-atom and the substituent’s OH group established four conventional bonds; in compound **8k**, the thiadiazole ring’s S-atom, along with the substituent’s F-atom, formed two HBs, while compounds **8b** and **8e** exhibited one conventional-H bond through the thiadiazole ring’s N-atom formed and the substituent’s CN moiety, respectively. Furthermore, aromatic amino acid interactions, such as π-π T-shaped, π-π stacked, π-sulfur, π-alkyl, and π-lone pair, performed a dynamic role in maintaining the ligands within the binding pocket, emphasizing the importance of aromatic and hydrophobic pharmacophoric factors. In compound **8k**, the substituent’s F-atom engaged the crucial residue ASP69 via halogen interaction, contributing to electrostatic stabilization. Massive van der Waals contacts display efficient pocket filling and advantageous hydrophobic complementarity. Collectively, compound **8g**’s inhibitory activity (*IC*_50_ = 7.60 ± 0.80 µM) against α-glucosidase is supported by its low docking score (−7.396 kcal/mol), representing strong binding affinity and stable interactions with key active-site residues. Furthermore, compound **8k** (*IC*_50_ = 8.42 ± 0.90 µM) was ranked second, showing a docking score value of −7.175 kcal/mol, **8b** (*IC*_50_ = 10.32 ± 0.90 µM) as third (binding affinity = −6.615 kcal/mol), and **8e** (*IC*_50_ = 14.75 ± 1.30 µM) as fourth (binding affinity = −6.595 kcal/mol). This validates computational calculations and strengthens their potential as enzyme inhibitors (Table 3).

### 2.4. DFT Analysis

#### 2.4.1. Frontier Molecular Orbitals (FMOs) and Band Gap

The electronic properties of the synthesized compounds (**8g**, **8k**, **8b**, and **8e**) were calculated using density functional theory (DFT) and analyzed in terms of their frontier molecular orbitals (FMOs). The HOMO energy is the electron-donating potentiality of a molecule, and the LUMO energy is the electron-accepting potentiality of a molecule.

The HOMO energies range from −5.544 to −6.326 eV, and the LUMO energies are −1.983 to −2.647 eV, as shown in Table 4. Among the studied compounds, the HOMO energy of **8g** is the highest (−5.544 eV), indicating a greater propensity to donate electrons. Conversely, compound **8e** is the least reactive to HOMO (−6.326 eV), which implies higher stability and reduced donating power of electrons. The energy gap ΔE was calculated as ΔE = E_LUMO_ − E_HOMO_. One of the main parameters to consider when determining chemical reactivity and kinetic stability is E_HOMO_. The obtained band gaps range from 3.563 to 3.733 eV. Compound **8g** has the smallest band gap (3.563 eV), which means that it is more chemically reactive and less kinetically stable, while compound **8k** has the largest band gap (3.733 eV), which means that it is relatively more stable and less reactive. Biologically, the decrease in the energy gap for compound **8g** may promote increased electronic polarization and intermolecular charge transfer interactions with the amino acid residues of the active site of the α-amylase and α-glucosidase enzyme, thus accounting for its better docking profile and enzyme inhibitory activity. Conversely, the relatively large band gap of compound **8k** might help to stabilize the electrons while still retaining good binding interactions in the catalytic pocket. These results indicate that the proper balance of molecular reactivity and stability might have a supportive role in the modulation of the enzyme–ligand interactions. Overall, the band gap trend indicates that the compound with the highest reactivity is **8g**, whereas the compound with relatively high stability is **8k**.

Although these electronic descriptors derived from DFT do not directly form the enzyme inhibition, they offer valuable information on the electronic behavior and reactivity of the synthesized derivatives, which may lead to the favorable interaction propensity of the synthesized derivatives in the active site observed in molecular docking studies.

#### 2.4.2. Reactivity Indices and Chemical Behavior

In order to gain further insight into the chemical behavior of the synthesized derivatives, global reactivity descriptors were computed by use of Koopmans. The ionization potential (IP) and electron affinity (EA) indicate a molecule’s tendency to gain or lose electrons, respectively. Compound **8e** has the greatest IP (6.326 eV), which means that it is most resistant to the removal of electrons and most stable, and compound **8g** has the least IP (5.544 eV), which means that it is comparatively easy to remove electrons. Likewise, the EA values rise from 1.983 eV (**8g**) to 2.647 eV (**8e**), indicating an upward trend in the tendency to accept electrons in the series. Electronegativity (*χ*) and chemical potential (*µ*) also explain the electronic distribution of the molecules. Compound **8e** has the greatest electronegativity (4.487 eV) in that it is highly electronegative and, therefore, very likely to attract electrons. The chemical potential (−4.487 eV) of the compound is very high, and therefore, the electronic configuration of the compound is stable. Conversely, the compound **8g** has the lowest electronegativity (3.764 eV), which is consistent with its greater reactivity. The important indicators of molecular stability and reactivity are global hardness (*ƞ*) and softness (*S*). Compound **8k** has the maximum hardness (1.867 eV), implying that it is more resistant to charge transfer and more stable, and compound **8g** has the minimum hardness (1.781 eV), implying that it is more reactive chemically. The softness values are inversely related, with **8g** being the softest molecule, again supporting its increased reactivity.

**Figure 4:**
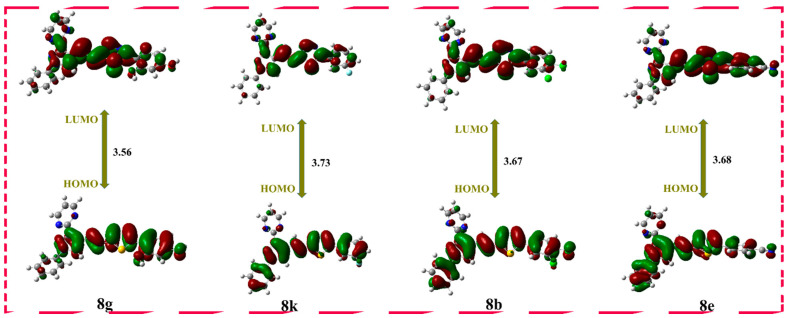
The calculated ground state isodensity surface plots for frontier molecular orbitals (FMOs) for investigated compounds **8g**, **8k**, **8b**, and **8e**.

The electrophilicity index (*ω*) is used to characterize the stabilization energy of a system upon gaining an extra electron. The most electrophilic compound is **8e**, with a value of 5.467 eV, and it is strongly electrophilic. In contrast, the least electrophilic compound is **8g**, with a value of 3.977 eV, and it is relatively weakly electrophilic. In general, the results of the DFT indicate that compound **8g** is the most reactive and the softest one, and compound **8e** is the most electrophilic and electronically stable. Compound **8k** is the hardest and has the largest band gap, which means it is comparatively more kinetically stable than the other derivatives studied (Figure 4).

#### 2.4.3. Molecular Surface and Electrostatic Potential (ESP) Maps

The maps of molecular electrostatic potential (ESP) were created to observe charge distribution and define the electrophilic and nucleophilic regions of the compounds produced after synthesis (**8g**, **8k**, **8b**, and **8e**). The ESP surface is overlaid onto the electron density isosurface, with different colors indicating different electrostatic potentials. The red areas indicate electron-rich areas (negative potential), and the blue areas indicate electron-deficient areas (positive potential). In-between colors, like green, depict neutral potential areas.

**Figure 5:**
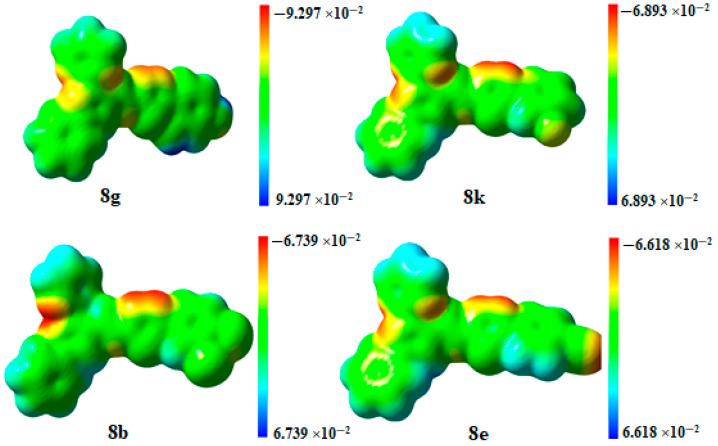
The ESP maps for investigated compounds **8g**, **8k**, **8b**, and **8e**.

Figure 5 indicates that negative potential areas (red) are mainly concentrated on electronegative atoms, such as oxygen, nitrogen, and sulfur, found in the oxadiazole, triazole, and thiol groups. These areas are potential sites of electrophilic interactions and play an important role in forming hydrogen bonds with amino acid residues in the enzymes’ active sites. The positive potential regions (blue), on the other hand, are predominantly spread around the hydrogen atoms and some aromatic regions, suggesting potential sites of nucleophilic interactivity. The electrostatic potential maps indicate that the heteroatoms present in the molecular structure play a major role in delocalizing charge, thereby contributing to greater interaction with the compounds’ biological targets. Interestingly, compounds with stronger negative electrostatic regions exhibit enhanced electron density distribution that may facilitate favorable non-covalent interactions with amino acid residues in the enzyme active sites, consistent with the interaction patterns observed in molecular docking studies (Table 5).

### 2.5. Molecular Dynamics Simulation

Molecular dynamics simulations were conducted specifically for compound **8g** because it demonstrated the most potent dual inhibitory activity against α-amylase and α-glucosidase, together with the most favorable docking scores and interaction profiles among the synthesized derivatives. Therefore, this compound was selected as the representative lead candidate to evaluate the dynamic stability and persistence of ligand–protein interactions under simulated physiological conditions.

**Figure 6:**
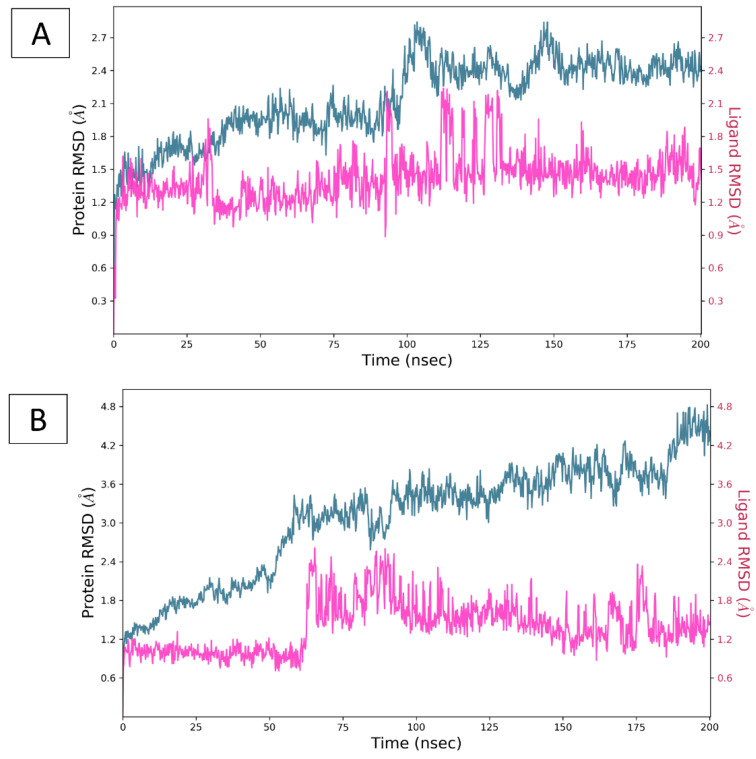
The root mean square deviation between C-alpha atoms and lead compound. (**A**) α-Amylase_**8g** complex and (**B**) α-Glucosidase_**8g** complex.

Molecular dynamics simulation of 200 ns was conducted to investigate the behavior of selected complexes including α-Amylase_8g complex and α-Glucosidase_**8g** complex. RMSD, RMSF, protein–ligand contacts, and SSE trajectories were analyzed to verify the stability of ligands within binding pockets certain of proteins. The RMSD analysis of α-Amylase_**8g** complex demonstrates that the protein RMSD increased initially due to the structural relaxation within the simulation environment (Figure 6A). The sharp rise at 100 ns was observed where RMSD fluctuates around 1.9 Å to 2.7 Å, suggesting conformational changes within the protein. After 150 ns, the protein RMSD remains stable at approximately 2.4 Å as protein is considered equilibrated in this new conformation. The ligand RMSD remains consistent around 1.2 Å and 1.8 Å for most of the time of simulation. Notably, the protein undergoes changes at 100 ns, so the ligand RMSD also increased around 2.2 Å between 100 ns and 130 ns. This indicates that the ligand adjusts itself during protein’s movement. After 130 ns, the ligand again settles itself within protein’s binding pocket. Overall, the data suggest that the results of the later phase (after 150 ns) are reliable for further analysis. Overall, the results suggest stable dynamic behavior.

The RMSD trajectory of α-Glucosidase_**8g** complex demonstrates that protein RMSD increases gradually around 1.2 Å to 3.0 Å for the first 50 ns, indicating conformational adjustment (Figure 6B). The significant sharp peaks observed at 60 ns and 185 ns indicates that protein changes its conformational states rather than fluctuating within a single conformation. The ligand remains bounded when protein shows maximum volatility. The ligand remains dynamically stable around 1.8 Å for 100 ns to 200 ns.

**Figure 7:**
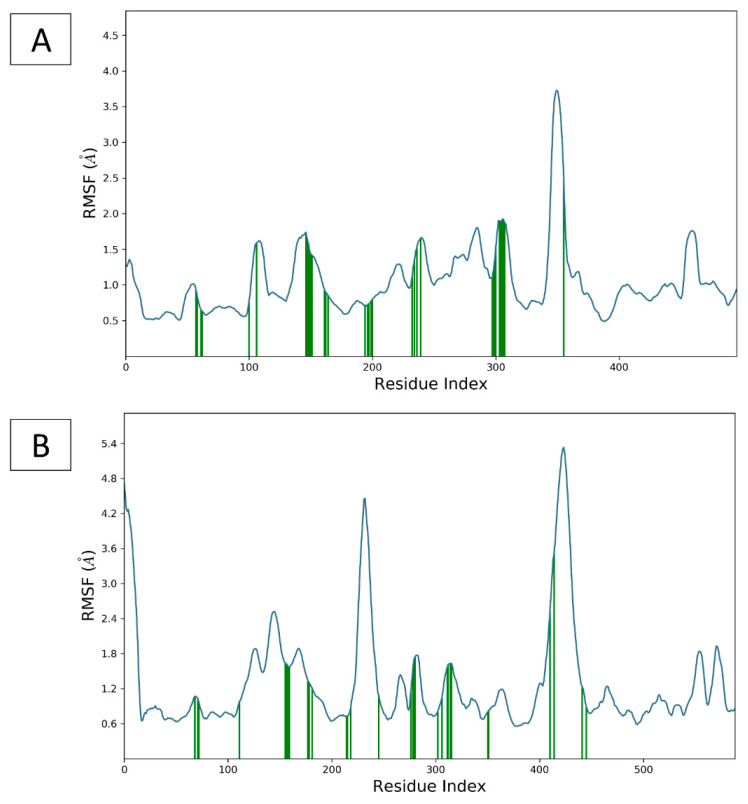
The root mean square fluctuation analysis over 200 ns of simulation. (**A**) α-Amylase_**8g** complex and (**B**) α-Glucosidase_**8g** complex.

The flexibility of individual residue has been observed through RMSF trajectory, highlighting the most mobile residues by sharp peaks. The average RMSF of α-Amylase_**8g** complex remains below 1.5 Å for most of the residues during 200 ns of simulation (Figure 7A). The residues showing the most significant peaks are ASN350, GLY351, and ASN352, having RMSF values greater than 5 Å. The secondary peaks indicate the ligand adjusts itself within the binding pocket. The green bars are associated with low RMSF areas indicating the stable binding pocket.

The RMSF plot of α-Glucosidase_**8g** complex, showing RMSF values predominantly below 1.5 Å, indicating the structural stability of protein’s secondary structure elements throughout the simulation (Figure 7B). The peaks on the RMSF plots, which are obviously high, correspond to the N- and C-terminal tails and peripheral loops that are not involved with ligand binding but have higher degrees of mobility. The residues showing the sharp peaks are MET 1, THR 2, THR 10, VAL 232, ASP 233, GLU 421, GLU 422, HIS 423, GLY 424, GLU 425, ASN 426, and SER 427, having RMSF values greater than 5 Å. The green bars are located within areas of low to moderate RMSF, indicating a stable ligand binding environment.

**Figure 8:**
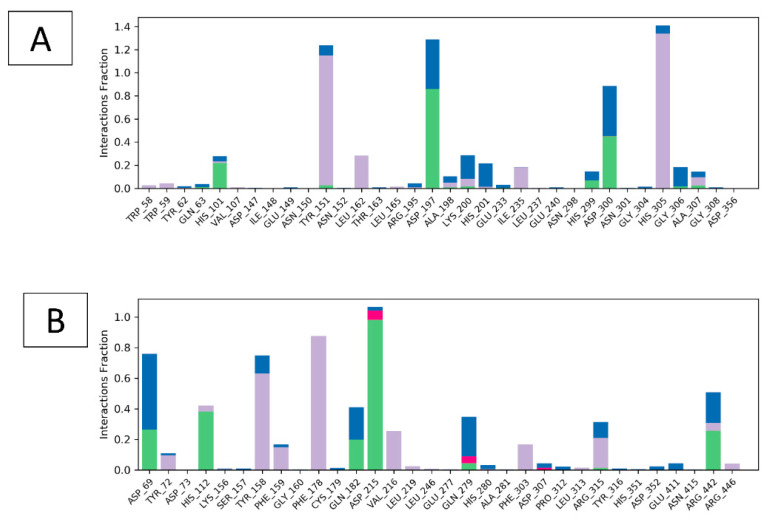
Protein–ligand contact histogram displays the interaction fraction and type of chemical bonds between ligand and protein residues. The green bars representing the hydrogen bonds, purple bars indicating hydrophobic contacts, blur bars representing water bridges and pink bars denote ionic or polar interactions. (**A**) α-Amylase_**8g** complex and (**B**) α-Glucosidase_**8g** complex.

The protein–ligand contact histogram gives deep insight into the type of chemical bond and interaction fraction over 200 ns of simulation. The α-Amylase_**8g** complex was primarily stabilized by making a hydrogen bond with ASP 197 and ASP 300 and **a** water-mediated hydrophobic bond with HIS 305 and TYR 151 (Figure 8A). The α-Glucosidase_**8g** complex was primarily stabilized by a consistent hydrogen bond with ASP 215 and hydrophobic bond with TYR 158 and PHE 178 (Figure 8B). The interaction fraction suggests that these residues are the major anchor of ligand stability.

**Figure 9:**
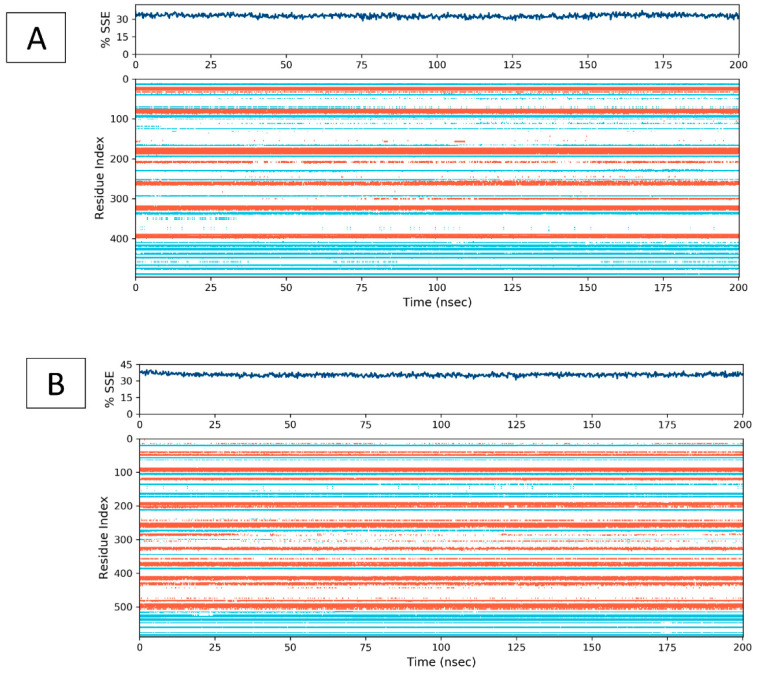
Distribution of protein secondary structure element over 200 ns of simulation. The horizontal red lines representing alpha-helices and blue lines indicating beta-strands. (**A**) α-Amylase_**8g** complex and (**B**) α-Glucosidase_**8g** complex.

The secondary structure timeline of α-Amylase_**8g** complex demonstrates that 35% of protein residues are a part of the protein’s secondary structure form, of which 22.25% is made up of helices and 13.27% is made up of beta sheets (Figure 9A). The SSE timeline of α-Glucosidase_**8g** complex illustrates that the protein consists of 35.52% of secondary structure, of which 22.25% is made up of alpha helices and 13.27% is made up of beta sheets (Figure 9B). The few regions in the bottom panel showing the discontinuous horizontal bars indicate transitions in structural states. Overall, proteins maintain their native fold over 200 ns of simulation.

**Figure 10:**
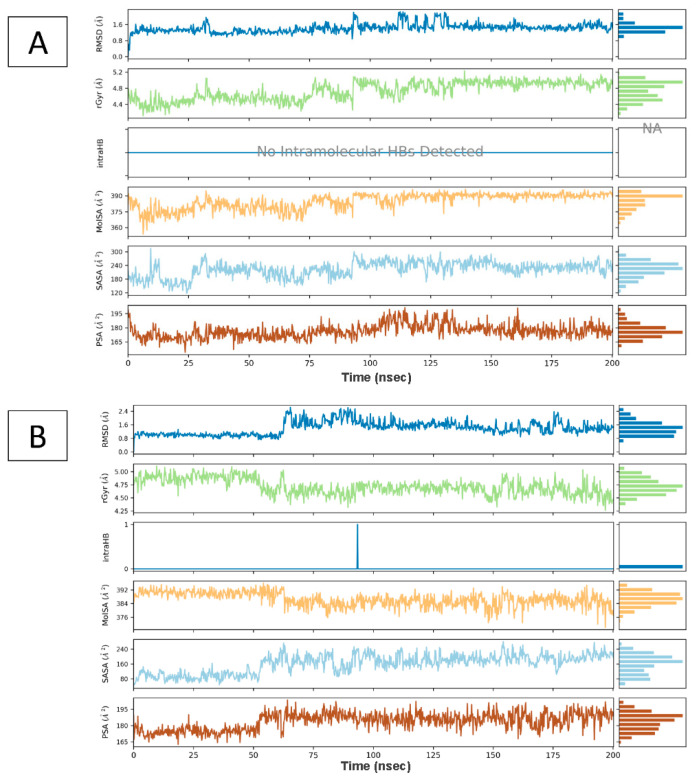
The assessment of ligand properties including RMSD, rGyr, intramolecular hydrogen bonds, MolSA, SASA, and PSA over 200 ns simulation. (**A**) α-Amylase_**8g** complex and (**B**) α-Glucosidase_**8g** complex.

The ligand property plots of α-Amylase_**8g** complex illustrate the ligand binding and stable behavior over 200 ns of simulation (Figure 10A). The ligand RMSD plot demonstrates that the ligand fluctuates initially around 1.5 Å. The results suggest that ligands explore different orientations before stabilization. Overall, the RMSD below 2 Å indicates the stability of the ligand within protein’s binding site. The rGyr plot is used to observe the extendedness of the molecule. The rGyr values remain between 4.4 and 5.0 Å. The rGyr mirrors the behavior of RMSD at 90 ns, suggesting the ligand orientation and extendedness within the binding site. The ligand made no intramolecular hydrogen bond. The MolSA values fluctuate between 360 Å^2^ and 390 Å^2^, indicating the ligand is highly flexible. SASA values fluctuate between 120 Å^2^ and 300 Å^2^, showing high volatility, suggest the binding pocket allows water molecule interaction with the ligand periodically. The PSA profile is relatively consistent, which indicates that the polar atoms interact with residues over 200 ns of simulation. Overall, the results suggest dynamic stability of the ligand.

The ligand property plots of α-Glucosidase_**8g** complex illustrate the ligand binding and dynamic behavior over 200 ns of simulation (Figure 10B). The ligand RMSD plot shows that the ligand shifted from a docked pose to a more energetically favorable pose. The rGyr decreased from 4.9 Å to 4.5 Å, indicating that the ligand adopted a more compact conformation during simulation time period. The MolSA value remained high during the initial 60 ns, then decreased subsequently, suggesting the ligand adopted a more compact conformation. The formation of intramolecular hydrogen bonds indicating the stabilization of ligand internal geometry. SASA increases after 60 ns, indicating that the ligand becomes extended with new conformation. PSA mirrors the behavior of SASA and stabilizes after 60 ns at a higher value.

**Figure 11:**
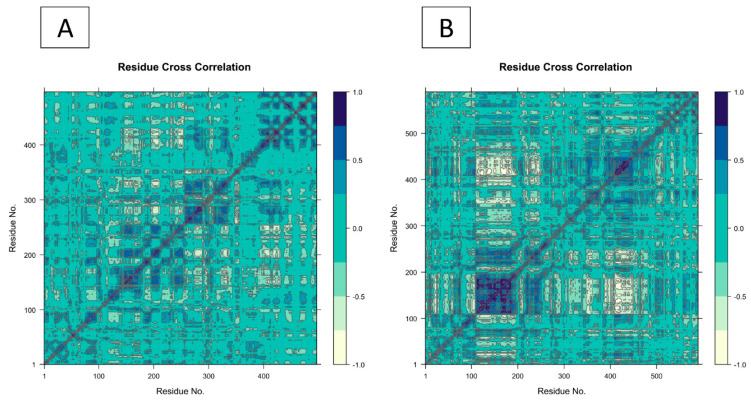
Dynamic cross-correlation map depicts the positive and negative correlation of residues. (**A**) α-Amylase_**8g** complex and (**B**) α-Glucosidase_**8g** complex.

A dynamic cross-correlation map (DCCM) is used to investigate the positive correlation and negative correlation of protein residues. The positive correlation is represented by dark blue, the negative correlation is represented by aquamarine color, and 0 correlation is shown by light sea green. The DCCM analysis of α-Amylase_**8g** complex reveals the maximum positive correlation with limited negative correlation, indicating the protein is dynamically stable with no major unfolding (Figure 11A). Additionally, the DCCM analysis of α-Glucosidase_**8g** complex illustrates a strong positive correlation along with negative correlation, indicating the protein flexibility of the protein (Figure 11B).

**Figure 12:**
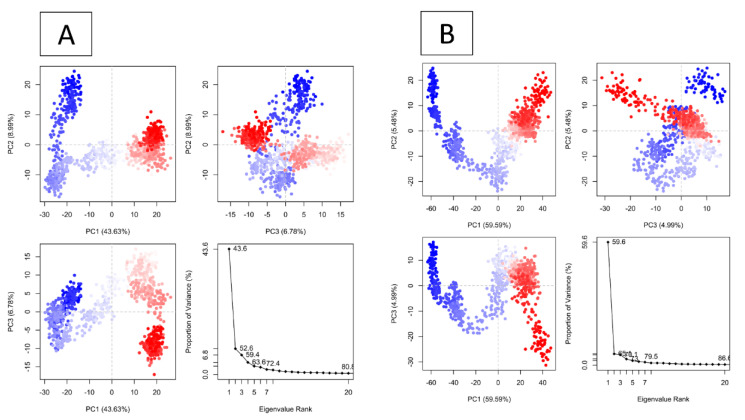
The PCA captures the high flexibility of residues. (**A**) α-Amylase_**8g**complex and (**B**) α-Glucosidase_**8g** complex. The color gradient representing the simulation progression which is gradually changing from dark blue to white and eventually to red clusters over time.

The principal component analysis (PCA) is used to detect the dynamic nature of α-Amylase_**8g** complex and α-Glucosidase_**8g** complex. The scree plot maps eigenvalues against their corresponding eigenvector index for the first 20 principal components and characterizes the system’s essential dynamics, indicating overall flexibility of the structures. The highest eigenvalues for α-Amylase_**8g** complex ranged from 43.6% to 72.4%. The major drivers of mobility were PC1 (43.63%), followed by PC2 (8.99%) and PC3 (6.78%) (Figure 12A). The highest eigenvalues for α-Glucosidase_**8g** complex ranged from 59.6% to 79.5%. The highest mobility was captured by PC1 (59.59%), followed by PC2 (5.48%) and PC3 (4.99%) (Figure 12B). The results suggest that PC3 had more rigid structure than PC1 and PC2 and is thought to have a more consistent binding site. The conformational variation across all groups is represented by blue, white, and red colors, representing high mobility, moderate mobility, and less mobility, respectively.

The binding free energy of α-Amylase_**8g** complex and α-Glucosidase_**8g** complex was calculated by the MMGBSA technique. The docking score of α-Amylase_**8g** complex was −7.222 kcal/mol, and for α-Glucosidase_**8g** complex, it was −7.396 kcal/mol. These results were verified by MMGBSA as it uses a more accurate scoring function. Snapshots were taken after every 20 ns from 200 ns molecular dynamics trajectory. The average binding energy of α-Amylase_**8g** complex was −63.27168529 kcal/mol, and for α-Glucosidase_**8g** complex, it was −63.5733064 kcal/mol. The binding energies of α-Amylase_**8g** complex and α-Glucosidase_**8g** complex are shown in Tables S1 and S2 in the Supplementary Information File.

Overall, the simulation results of α-Amylase_**8g** complex and α-Glucosidase_**8g** complex showed dynamic stability. Comparatively, the α-Amylase_**8g** complex is more stable, and the ligand was more stable within the binding site by making multiple chemical bonds and showing more consistent profiles. However, the **8g** compound is a potential inhibitor, but further analyses are required for its usage as a medicine (Figure 12).

Although MD simulations were not extended to acarbose and the remaining derivatives, comparative molecular docking analysis already established the relative binding affinities and interaction patterns of all tested compounds. The MD investigation was therefore focused on validating the dynamic behavior of the most biologically active derivative, **8g**, within the catalytic pockets of the target enzymes.

### 2.6. ADMET Analysis

#### 2.6.1. Physicochemical Properties

Physicochemical characteristics of the synthesized products (**8g**, **8k**, **8b**, and **8e**) were measured to assess their drug-likeness and oral bioavailability. All compounds have a molecular weight of between 427.46 and 478.36 g/mol, which is not too much out of the acceptable range of drug-like molecules. The number of hydrogen-bond donors (0–2) and acceptors (6–8) is also within acceptable limits, indicating that it is likely to interact with biological targets. The TPSA values range from 109.98 to 150.44 Å^2^, indicating moderate polarity. Not only Compound **8k** and **8b**, which have lower values of TPSA (approximately 110 Å^2^), should have better membrane permeability than Compound **8g** and **8e**. Moreover, each compound has a low proportion of sp^3^ carbons, making them highly aromatic and associated with their high binding affinity for the enzyme’s active site.

#### 2.6.2. Pharmacokinetic Properties

The pharmacokinetic profile indicates that compound **8k** exhibited high gastrointestinal (GI) absorption, whereas the other compounds (**8g**, **8b**, and **8e**) exhibited low absorption, which could limit oral bioavailability. All the compounds are unlikely to cross the blood–brain barrier (BBB), and side effects on the central nervous system are unlikely. It is expected that most compounds are substrates of P-glycoprotein (P-gp), suggesting efflux-related restriction of drug accumulation. Notably, none of the compounds inhibit major cytochrome P450 enzyme groups, such as CYP1A2, CYP2C19, and CYP2D6, implying fewer risks of drug–drug interactions. Nevertheless, they all inhibit CYP2C9, and compound **8e** also inhibits CYP3A4, which could alter metabolic stability and warrants further research.

#### 2.6.3. Drug-Likeness Evaluation

**Figure 13:**
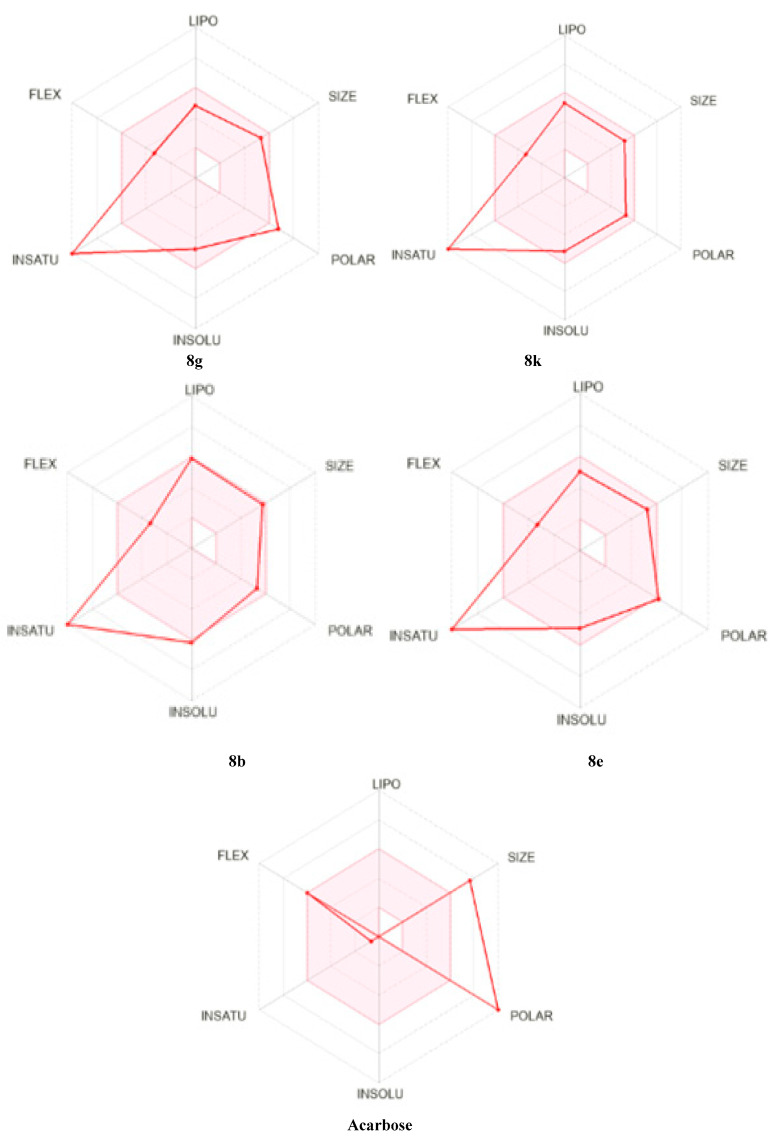
Drug-likeness evaluation of target compounds and acarbose.

Most of the rules, such as Lipinski, Ghose, Veber, Egan, and Muegge filters, were used to determine drug-likeness. The investigated compounds, **8k**, **8b**, and **8e**, are good drugs, as most of these rules are met. Conversely, compound **8g** does not meet many of the criteria, probably because it is more polar and can form hydrogen bonds. The bioavailability score is 0.55 across all compounds, much higher than the oral bioavailability of the standard drug acarbose (0.17), indicating greater potential for oral bioavailability. Additionally, none of the derivatives have PAINS alerts, indicating that the compounds are unlikely to cause false-positive biological reactions. The physicochemical predictions, pharmacokinetics, drug-likeness, and medicinal chemistry of the derivatives under study also support this conclusion (Figure 13).

#### 2.6.4. Toxicological Assessment

ProTox-II toxicity predictions indicate that all synthesized compounds are in toxicity class 4 (moderate toxicity), whereas acarbose is in toxicity class 6 (non-toxic). The estimated LD50 values of the synthesized compounds were predicted to be 1000 mg/kg, indicating moderate acute toxicity. However, the simultaneous prediction of hepatotoxicity, carcinogenicity, and mutagenicity for some derivatives highlights potential safety concerns that warrant careful consideration and further experimental toxicological validation. All compounds are predicted to be hepatotoxic and may require structural optimization. Most compounds, except **8k**, are carcinogenic, with **8g** and **8b** predicted to be mutagenic. Only compound **8b** has significant immunotoxicity. Although the compounds were predicted to be non-cytotoxic, the observed toxicity alerts suggest that additional optimization may be necessary to improve the overall safety profile of these derivatives (Table 6).

#### 2.6.5. StopTox Analysis

Predictions of the compounds using the StopTox online web server tool also confirm their safety profile, with a moderate likelihood of acute oral, dermal, and inhalation toxicity. Sensitization and irritation values for the skin are acceptable and indicate manageable levels of toxicity. The StopTox predictions suggest moderate toxicity profiles for the synthesized derivatives; however, the presence of predicted hepatotoxicity- and genotoxicity-related endpoints indicates that these compounds should be considered preliminary leads requiring further optimization and detailed biological safety evaluation (Table 7).

## 3. Materials and Methods

### 3.1. Chemicals and Instruments

All chemicals and reagents required were purchased from Sigma-Aldrich (St. Louis, MO, USA) and used without further purification. The progress of the reactions and preliminary verification of the synthesized compounds were monitored by thin-layer chromatography (TLC). ^1^H and ^13^C NMR spectra were recorded on a Bruker Avance III spectrometer (Bruker BioSpin GmbH, Rheinstetten, Germany) operating at 500 MHz for ^1^H and 125 MHz for ^13^C nuclei, with tetramethylsilane (TMS) employed as the internal reference standard. Chemical shifts (δ) are expressed in parts per million (ppm), while coupling constants (*J*) are reported in hertz (Hz). High-resolution mass spectrometric analyses were carried out using an electrospray ionization (ESI) source coupled to an AB SCIEX Q-TOF mass spectrometer (AB SCIEX, Concord, ON, Canada). The measured m/z values of the corresponding adduct ions were compared with their calculated exact masses for structural confirmation.

### 3.2. In Vitro Assays

#### 3.2.1. Alpha-Amylase Activity Assay

The inhibition potential of α-Amylase enzyme was assessed using standard techniques [31]. A test tube with 250 µL of the chemical being evaluated at varied concentrations (50–250 µg/mL), [1% (*w*/*v*)] starch solution, and (1 U/mL) alpha-amylase solution was created. After 3 min of incubation at 20 °C, the enzymatic reaction was interrupted by 500 µL of dinitro salicylic acid (color reagent). After boiling the mixture in hot water, 250 µL of α-amylase was added immediately. The mixture was heated to 85 °C for 15 min, then incubated for 5 min at room temperature. Distilled water (4500 µL) was added to reach a final volume of 6000 µL. Absorbance was determined using a spectrophotometric analysis at 540 nm. A control sample without the test ingredient was generated, and acarbose was used as the reference medication. The percentage inhibition equation is utilized for computation.

#### 3.2.2. Alpha-Glucosidase Activity Assay

Alpha-glucosidase’s inhibitory potential was tested using a well-established approach [31]. In a 96-well plate, each well received 35 µL of phosphate buffer, 31 µL of the tested chemical solution (concentration range: 50–250 µg/mL), and 18 µL of [4-nitrophenyl-α-D glucopyranoside (p-NPG)] substrate. The mixture was then incubated at 37 °C for 5 min. Then, 16 µL of α-glucosidase (0.15 U/mL) in sodium phosphate was added to each well, for a total volume of 100 µL. The reaction began with the addition of 100 µL of sodium carbonate (200 mM). The absorbance at 405 nm was measured with a microplate reader. The experiment was repeated three times using a control group that did not receive the tested substance for comparison purposes. Acarbose was used as the standard reference medication. The % inhibition was computed using the equation below, where “Abs” denotes absorbance.

% inhibition = [(Abs Control − Abs Sample)/Abs Control] × 100

### 3.3. Docking Methodology

The binding orientations, affinities, and interaction profiles of the synthesized compounds with the target enzymes 1B2Y(alpha-amylase) and 3A4A(alpha-glucosidase) were investigated by molecular docking studies using AutoDock Tools (version 1.5.7), AutoDock Vina, and Biovia Discovery Studio Visualizer 2021. RCSB Protein Data Bank was used to retrieve protein structures [32,33]. The re-dock method was utilized to validate the docking protocol prior to inserting the target compounds into the binding sites.

The inhibitors’ chemical structures were initially sketched using ChemDraw 22.0.0 software. Geometry optimization and energy minimization were then carried out. To make all ligand structures compatible with AutoDock Vina, all ligand structures were generated and transformed into PDBQT format using AutoDock Tools. After preparing the ligand, the protein was extracted from PDB and subjected to pre-docking optimization, which included assigning Kollman charges, adding hydrogen atoms, and eliminating water molecules. The binding site was located according to the location of the co-crystallized ligand in each protein structure. In particular, the grid box center was calculated as the geometric center of the native ligand, with the grid dimensions adjusted to cover the active site residues as well as provide enough ligand flexibility.

For α-amylase (PDB ID: 1B2Y), the grid box was centered at coordinates X = −21.45, Y = 47.32, and Z = 18.76. In the case of α-glucosidase (PDB ID: 3A4A), the grid center was set at X = 22.731, Y = −8.34, and Z = 20.661. Moreover, to adequately cover the binding regions of interest, a blind docking strategy was adopted by increasing the grid box to cover the whole protein structure. Using evolutionary algorithm configurations with 10 runs and 2,500,000 energy assessments per run, docking parameters were generated. The scoring function used kcal per mole as the unit, and a lower score corresponded to an appropriate active-site alignment. Pymol and BIOVIA Discovery Studio Client 2021 were used to evaluate the nonbonding interactions between the docked protein–ligand complexes and the docking pose [34,35].

### 3.4. DFT Methodology

Density functional theory (DFT) was used to calculate the electronic properties and reactivity of the compounds synthesized (**8g**, **8k**, **8b**, and **8e**). The ground-state geometries of the molecular structures were obtained by first building them and then optimizing them without symmetry constraints. The B3LYP functional with the 6-31G(d,p) basis set was used to perform geometry optimizations; the latter basis set has been extensively used to provide dependable predictive capabilities for molecular electronic properties. Calculations of frequencies were also carried out at the same level of theory to ensure that the optimized structures are true minima of the potential energy surface, as indicated by the absence of imaginary frequencies. The optimized structures yielded the frontier molecular orbital (FMO) energies, as well as the highest occupied molecular orbital (HOMO) and lowest unoccupied molecular orbital (LUMO) energies. The chemical stability and reactivity of the molecules were determined by the calculation of the energy gap (DE) between HOMO and LUMO. The global reactivity descriptors were calculated by Koopmans theorem, in which the ionization potential (IP) and electron affinity (EA) were estimated as the negative of HOMO and LUMO energies, respectively. According to these values, other descriptors like electronegativity (χ), chemical potential (*µ*), global hardness (*ƞ*), and global softness (*S*), as well as electrophilicity index (*ω*), were computed by means of standard equations:

- $\mathrm{IP}=-E_{\mathrm{HOMO}}$
- $\mathrm{EA}=-E_{\mathrm{LUMO}}$
- $\chi =\frac{\mathrm{IP}+\mathrm{EA}}{2}$
- μ=−χ
- $\eta =\frac{\mathrm{IP}-\mathrm{EA}}{2}$
- $S=\frac{1}{2\eta }$
- $\omega =\frac{\mu^{2}}{2\eta }$

In addition, molecular electrostatic potential (ESP) maps were created to show charge distributions and identify electrophilic and nucleophilic regions on the molecules. The ESP surfaces were overlaid on the electron-density isosurface with negative potential (red) and positive potential (blue), indicating sites rich in and deprived of electrons, respectively. All computations were performed in the gas phase, and the results were compared with the known chemical reactivity and stability of the synthesized derivatives to correlate the electronic structure [34,36].

### 3.5. Molecular Dynamics Simulations

The dynamic behavior of α-amylase (PDB ID: 1B2Y) and α-Glucosidase (PDB ID: 3A4A) in complex with **8g** compound was evaluated by MD simulation by using the Desmond module of Schrodinger LLC [37]. The initial coordinates were selected on the basis of molecular docking results. The systems for two complexes were prepared by using Maestro’s Protein Preparation Wizard tool [38]. An orthorhombic box of dimensions 10 × 10 × 10 Å was selected and solvated by using the TIP3P solvent model. The system was neutralized by adding 0.15 M concentration of counter ions (Na+ and Cl−) [39], and an OPLS_2005 force field was applied to mitigate the system [40]. Before the production phase, both solvated systems undergo the standard multistage relaxation protocol in Desmond to remove steric clashes. The system was energy minimized before the simulation run. The simulation was run at 310 K temperature and 1 atm pressure with NPT ensemble. The temperature was maintained by the Nose–Hoover chain thermostat, and pressure was controlled by the Martyna–Tobias–Klein barostat [41]. Additionally, the data concerning RMSD, RMSF, SSE, rGyr, and protein ligand contacts were collected to evaluate the complexes’ stability and dynamic behavior. PCA and DCCM were also computed by running R script with Bio3D package of R [42].

#### Binding Free Energy (M-GBSA) Calculation

The MMGBSA technique was used to evaluate the binding free energy of α-Amylase_**8g** complex and α-Glucosidase_**8g** complex. MMGBSA enables the validation of the results of molecular docking as it uses a more precise function of scoring [43]. The binding free energies were calculated by using the prime MM-GBSA module of Schrödinger under the force field of OPLS_2005, the VSGB solvent model, and rotamer search techniques applied to trajectory-derived conformations.

### 3.6. ADMET Methodology

The drug-likeness, bioavailability, and safety of the synthesized compounds (**8g**, **8k**, **8b**, and **8e**) were assessed using in silico ADMET prediction tools to evaluate their pharmacokinetics and toxicity. The chemical structures of all the compounds were drawn and converted to canonical SMILES, which were then used as inputs for computational analysis. The SwissADME web server was used to calculate physicochemical properties. The parameters were then used to assess the drug-likeness and oral bioavailability of the compounds using well-known rules. SwissADME was also used to predict pharmacokinetic properties and major cytochrome P450 (CYP) isoform inhibition. These parameters play a vital role in understanding drug absorption, distribution, metabolism, and potential drug–drug interactions. The toxicological measurements were performed using the ProTox-II online server, which estimates toxicity endpoints. The classification of toxicity classes was carried out using globally accepted standards, with classes 1–3 as high toxicity, classes 4–5 as moderate toxicity, and class 6 as non-toxic compounds.

Moreover, further toxicity assessment was performed using the StopTox platform, which predicts endpoints for acute toxicity. These parameters provide detailed information on the compounds’ safety profile. The suitability of the compounds for drug development was also assessed using SwissADME, which evaluated medicinal chemistry parameters. The lack of a PAINS warning was deemed a good indicator of trusted biological action, whereas synthetic accessibility scores were used to assess the compound’s synthetic simplicity. Every computation was performed under the default conditions of the corresponding web servers, and the calculated ADMET characteristics were used to correlate pharmacokinetic behavior with the observed biological activity of the synthesized compounds [44,45].

## 4. Conclusions

The current study focuses on the synthesis and evaluation of novel pyrimidine-derived pyrazole-based thiadiazole derivatives to target diabetes mellitus by inhibiting α-amylase and α-glucosidase. Twelve synthesized derivatives were structurally confirmed by utilizing ^1^H NMR and ^13^C NMR spectroscopy. The findings exhibited that, except for three compounds (**8i**, **8j**, and **8l**), all other synthesized derivatives inhibited α-amylase and α-glucosidase enzymes with *IC_50_* values ranging from 5.17 μM to 29.84 μM on α-amylase and 7.60 μM to 31.62 μM on α-glucosidase. Analogs **8g** (α-amylase *IC*_50_ = 5.17 ± 0.60 μM; α-glucosidase *IC*_50_ = 7.60 ± 0.80 μM), **8k** (α-amylase *IC*_50_ = 6.83 ± 0.70 μM; α-glucosidase *IC*_50_ = 8.42 ± 0.90 μM), and **8b** (α-amylase *IC*_50_ = 8.13 ± 0.80 μM; α-glucosidase *IC*_50_ = 10.32 ± 0.90 μM) displayed superior or comparable inhibitory activity compared to the reference drug acarbose (α-amylase *IC*_50_ = 8.25 ± 0.80 μM; α-glucosidase *IC*_50_ = 10.75 ± 1.10 μM. The SAR assessment underscores the importance of EDG hydroxyl at ortho and para positions, small EWG substituent fluorine at meta position, strong EWG substituent chlorine at meta and para positions, and their influence on the biological potential of the derivatives against both enzymes. The inhibitory potential of the derivatives is primarily supported by the in vitro enzyme inhibition assays together with favorable binding interactions and docking scores obtained from molecular docking and molecular dynamics analyses. In addition, DFT-based HOMO–LUMO calculations and electrostatic potential maps provided complementary insight into the electronic properties, charge distribution, and possible interaction behavior of the synthesized derivatives. According to ESP maps, all structures have two potential binding locations that the most positive and negative districts can conquer. The observed toxicity alerts in ADMET analysis suggest that additional optimization may be necessary to improve the overall safety profile of these derivatives. Overall, these findings specify that experimental outcomes and in silico validations display these revealed potential anti-diabetic properties and signify a pyrimidine-derived pyrazole-based thiadiazole as a promising scaffold for developing α-amylase and α-glucosidase inhibitors for the treatment of diabetic Mellitus.

## Figures and Tables

**Table 1:** Chemical structures, in vitro α-amylase, and α-glucosidase activity of synthesized compounds (**8a**–**8l**).

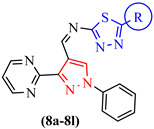					
Cpds	Chemical Structures	α-Amylase Inhibition *IC*_50_ ± SD (μM)	α-Glucosidase Inhibition *IC*_50_ ± SD (μM)	α-Amylase Selectivity ^a^	α-Glucosidase Selectivity ^b^
**8a**	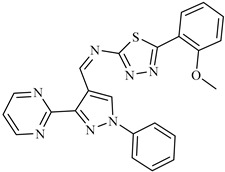	29.84 ± 2.10	31.62 ± 2.20	1.06	0.94
**8b**	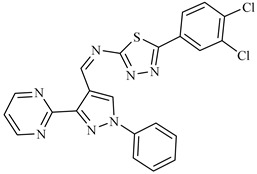	8.13 ± 0.80	10.32 ± 0.90	1.27	0.79
**8c**	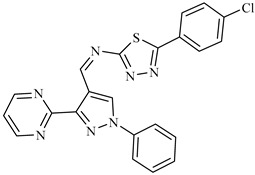	15.78 ± 1.30	17.49 ± 1.50	1.11	0.90
**8d**	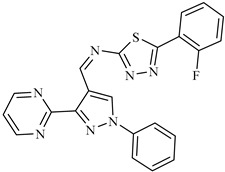	24.70 ± 1.80	27.39 ± 1.90	1.11	0.90
**8e**	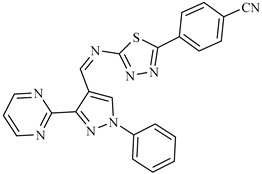	12.64 ± 1.20	14.75 ± 1.30	1.17	0.86
**8f**	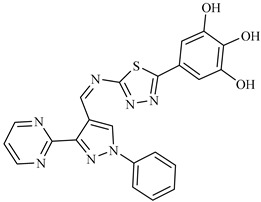	16.40 ± 1.60	18.78 ± 1.70	1.15	0.87
**8g**	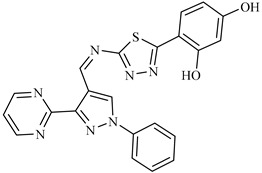	5.17 ± 0.60	7.60 ± 0.80	1.47	0.68
**8h**	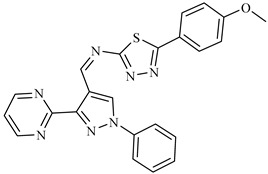	20.67 ± 1.80	22.13 ± 1.90	1.07	0.93
**8i**	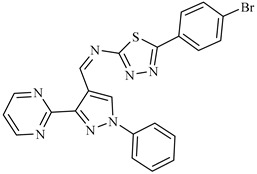	N.A	N.A	-	-
**8j**	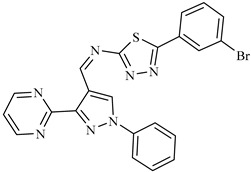	N.A	N.A	-	-
**8k**	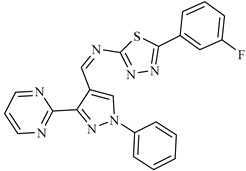	6.83 ± 0.70	8.42 ± 0.90	1.23	0.81
**8l**	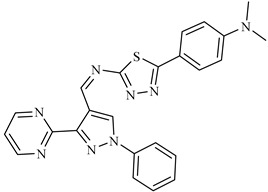	N.A	N.A	-	-
Standard Acarbose drug		8.25 ± 0.80	10.75 ± 1.10	1.30	0.77

^a^ α-amylase selectivity = *IC_50_* (α-glucosidase)/*IC_50_* (α-amylase). ^b^ α-glucosidase selectivity = *IC*_50_ (α-amylase)/*IC*_50_ (α-glucosidase). N.A stand for “Not Active”.

**Table 2:** Receptor residues with their type of interactions and bond distances of selected compounds and acarbose on α-amylase.

Comp. No.	RMSD (Å)	Conventional Hydrogen Bond	Bond Distance (Å)	Other Interacting Residues with Their Type of Interactions	Bond Distance (Å)	Van Der Waal Contacts
**Acarbose**	1.345	ASP300 THR163 TRP59 GLN63 GLU233	4.81, 5.41 3.15, 4.77 4.88 5.83 6.11	HIS301 (π-alkyl) THR163 (C-H) ASP300 (C-H) TRP59 (C-H) GLY104 (C-H) HIS201 (C-H)	6.36 4.46 4.69 3.55 3.81 5.19	TYR151, LYS200, ILE235, ASP197, GLY306, ARG195, HIS299, TRP58, TYR62, GLY164, VAL107, ILE51, LEU165, ALA198, LEU162
**8g**	0.876	GLU233 HIS305	3.23 3.59	HIS201 (π-π T-Shaped) TYR151 (π-π T-Shaped) LYS200 (π-alkyl) ALA198 (π-alkyl) ILE235 (π-alkyl) GLY306 (π-Donor-H) GLY304 (C-H)	5.43 6.91 5.89, 5.69 4.88, 6.23 6.07 3.70 5.07	SER199, TRP59, ASP300, VAL234, TYR62, LEU162
**8k**	1.023	HIS305 GLY306	4.09 4.20	HIS201 (π-π T-Shaped) ILE235 (π-alkyl) LYS200 (π-alkyl)	6.10 5.04 5.85	THR163, GLN63, TRP59, LEU165, TYR62, TRP58, HIS299, ARG195, ASP300, ASP197, GLU233, SER199, ALA198, VAL234, TYR151, LEU162
**8b**	0.978	LYS200	5.29	HIS201 (π-π T-Shaped) ILE235 (π-alkyl, π-alkyl) TYR62 (π-alkyl) LYS200 (π-alkyl) LEU162 (π-alkyl) ALA198 (π-alkyl) HIS299 (π-alkyl) TRP58 (π-alkyl) GLU240 (C-H)	5.77 4.39, 6.17 4.00 4.59 5.83 6.01 6.08 6.64 5.64	ARG195, ASP300, ASP197, TRP59, HIS305, GLY306, TYR151, LEU237, VAL234, SER199, THR163, GLU233
**8e**	1.276	GLN63	4.30	TYR151 (π-π Stacked) HIS201 (π-π T-Shaped) ILE235 (π-alkyl, π-alkyl) LYS200 (π-alkyl) ALA198 (π-alkyl) GLY306 (C-H)	5.04 5.99 4.49, 5.90 5.00 6.11 3.70	TRP59, TRP58, ASP300, HIS305, TYR62, GLU240, VAL234, GLU233, SER199, LEU162, THR163, LEU165,

**Table 3:** Receptor residues with their type of interactions and bond distances of selected compounds and Acarbose on α-glucosidase.

Comp. No.	RMSD (Å)	Conventional Hydrogen Bond	Bond Distance (Å)	Other Interacting Residues with Their Type of Interactions	Bond Distance (Å)	Van Der Waal Contacts
**Acarbose**	0.946	ASP307 THR306 ASP215 ASP69 ASP352 ARG315 ARG442	3.84 3.95 3.96 4.14 4.43 4.48 6.23	PHE303 (π-sigma) TYR158 (π-alkyl) VAL216 (Alkyl) PHE718 (π-alkyl) ASP352 (C-H, C-H, C-H) GLU277 (C-H, C-H, C-H) GLU411 (C-H, C-H, C-H) ARG442 (C-H) ASP307 (C-H) TYR72 (π-Donor-H) HIS112 (C-H) ASP215 (C-H) ASN350 (C-H) ASP352 (Unfavorable acceptor- acceptor)	2.98 4.48 5.10 6.05 4.64, 4.67, 5.06 3.78, 5.62, 6.21 4.83, 5.44, 5.69 4.50 4.87 2.97 4.89 4.98 3.77 3.62	PHE310, TRP58, TYR347, GLN353, TYR316, ARG446, PHE159, GLN182, VAL109, HIS351, LEU219, GLN279, ARG213, HIS280
**8g**	0.874	ARG442 HIS112 ASP69	4.98, 5.94 5.97 4.85	PHE303 (π-π Stacked) TYR72 (π-π T-Shaped) PHE159 (π-π T-Shaped) PHE178 (π-sulfur) VAL216 (π-alkyl) ARG315 (C-H, π-alkyl)	4.25 4.91 6.37 6.08 5.02 3.85, 4.37	VAL109, ARG213, ASP215, GLU411, TYR158, HIS280, PRO312, THR310, SER311, ASP307, GLN353, THR306, TYR347, ASN350, ASP352, GLU277, GLN182
**8k**	0.952	ARG442 GLN182	4.09 4.20	ASP69 (Halogen-F) PHE303 (π-π Stacked) TYR72 (π-π T-Shaped) PHE159 (π-π Stacked) ARG315 (π-alkyl) ASP307 (C-H) GLU411 (C-H)	4.92 4.47 4.68 6.00 4.39 4.70 6.14	VAL109, ASP215, TYR158, TYR316, HIS280, THR306, GLN353, TYR347, ASN350, PHE301, PHE178, ASP352, GLU277, HIS112, HIS351, ARG213
**8b**	1.431	ARG442	5.30	PHE303 (π-π Stacked, π-π Stacked) VAL216 (Alkyl, π-alkyl) TYR72 (π-alkyl) PHE178 (π-alkyl) HIS112 (π-alkyl) ARG315 (C-H, π-alkyl) HIS280 (C-H)	4.12, 6.35 4.02, 5.87 3.52 4.71 6.19 3.59, 4.34 6.25	ASP215, GLN279, GLU411, TYR158, THR310, SER311, PRO312, ASP307, THR306, TYR347, GLN353, ASN350, ASP352, GLU277, PHE159, HIS351, ARG446
**8e**	1.181	ARG213	5.88	PHE303 (π-π Stacked, π-π Stacked) PHE178 (π-π T-Shaped) TYR158 (π-Lone pair, π-π T-Shaped) ARG315 (π-alkyl)	4.23, 5.14 6.47 4.86, 5.14 5.65	TRP58, HIS351, ASP352, PHE301, THR306, GLN353, ASP307, LYS156, PHE314, ASN415, TYR316, GLU411, GLN279, PHE159, ARG442, VAL216, TYR347, ASN350, GLU277

**Table 4:** FMO energy of the synthesized hybrids.

Compound	HOMO (eV)	LUMO (eV)	Band Gap ΔE (eV)
**8g**	−5.54	−1.98	3.56
**8k**	−6.16	−2.42	3.73
**8b**	−6.22	−2.55	3.67
**8e**	−6.32	−2.65	3.68

**Table 5:** Reactivity indices of the synthesized derivatives.

Compound	IP (eV)	EA (eV)	χ (eV)	μ (eV)	η (eV)	S (eV^−1^)	ω (eV)
**8g**	1.98	5.54	3.76	−3.76	1.78	0.28	3.98
**8k**	2.42	6.16	4.29	−4.29	1.87	0.26	4.94
**8b**	2.55	6.22	4.38	−4.38	1.83	0.27	5.23
**8e**	2.65	6.32	4.49	−4.49	1.84	0.27	5.47

Abbreviations: Electron affinity, *EA*; ionization energy, *IP*; chemical softness, *S*; chemical hardness, *η*; chemical potential, *μ*; electronegativity, *χ*; electrophilicity, *ω*.

**Table 6:** Pro-Tox II toxicological parameters of the bioactive compounds.

Toxicity	8g	8k	8b	8e	Acarbose
Predicted LD50 (mg/kg)	1000	1000	1000	1000	24,000
Predicted Toxicity Class (Class 1–3: toxic, lethal if consumed; Class 4–5: moderate toxicity, harmful if consumed; Class 6: not toxic)	Class 4	4	4	4	6
Hepatotoxicity	Active (0.59)	Active (0.59)	Active (0.55)	Active (0.54)	Active (0.65)
Carcinogenicity	Active (0.60)	Inactive (0.55)	Active (0.58)	Active (0.58)	Inactive (0.84)
Immunotoxicity	Inactive (0.69)	Inactive (0.85)	Active (0.99)	Inactive (0.98)	Active (0.99)
Mutagenicity	Active (0.51)	Inactive (0.50)	Active (0.53)	Inactive (0.56)	Inactive (0.76)
Cytotoxicity	Inactive (0.76)	Inactive (0.80)	Inactive (0.55)	Inactive (0.84)	Inactive (0.70)

**Table 7:** StopTox toxicity parameters of the selected compounds.

Compound	Acute Inhalation Toxicity (%)	Acute Oral Toxicity (%)	Acute Dermal Toxicity (%)	Eye Irritation and Corrosion (%)	Skin Sensitization (%)	Skin Irritation and Corrosion (%)
**8g**	54	60	68	76	60	60
**8k**	63	53	51	71	60	70
**8b**	60	53	50	73	70	70
**8e**	64	53	54	68	50	80
**Acarbose**	63	96	86	58	70	70

## Data Availability

The original contributions presented in this study are included in the article. Further inquiries can be directed to the corresponding author.

## Supplementary Materials

The following supporting information can be downloaded at: https://www.mdpi.com/article/10.3390/ph19060915/s1. References [46,47] are cited in the Supplementary Materials.
