# Supplementary material for: Synthetic Elaboration, DFT Profiling, and Molecular-Dynamics-Guided Computational Validation Toward Anti-Diabetic Therapeutics: Tailored Pyrimidine-Derived Pyrazole-Thiadiazole Hybrid Scaffolds
Source: Pharmaceuticals (Basel). 2026 Jun 10;19(6):915. doi: 10.3390/ph19060915 (PMC13304519; doi:10.3390/ph19060915)
Supplement: Supplementary file 1 [file pharmaceuticals-19-00915-s001.zip › pharmaceuticals-4325726-supplementary.pdf]

Supplementary Information

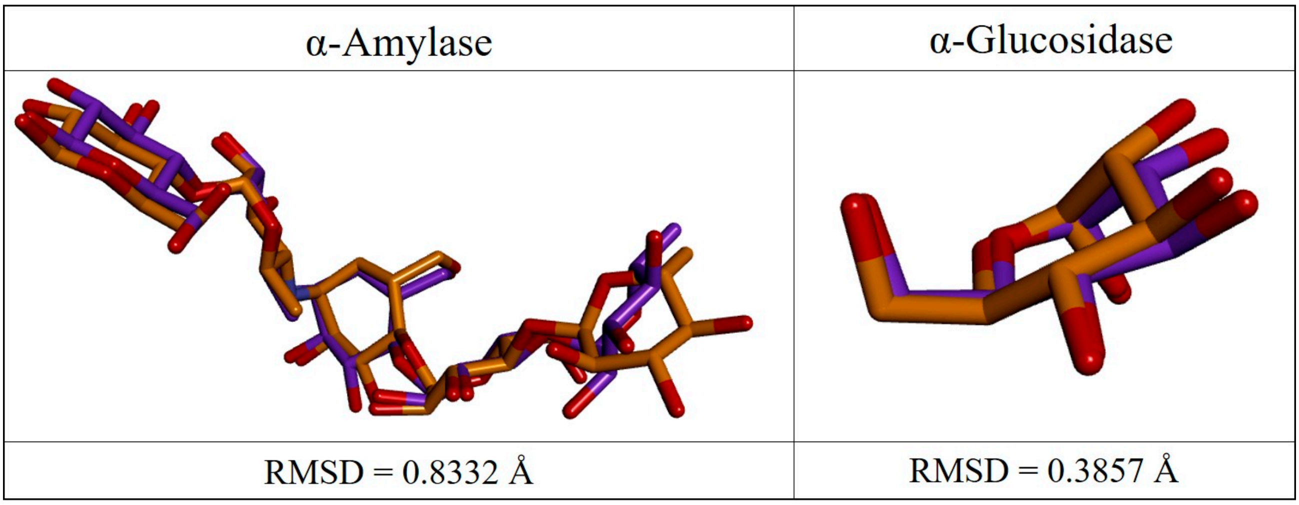

Figure S1: Superimposition of the native ligand pose and docked pose with the RMSD values for the binding sites of α-amylase and α-glucosidase.

Table S1. Binding free energies of α-Amylase_**8g** complex.

Time frame	dG_{bind}	dG_{b}Coulomb	$dG_{b}H_{bond}$
**0 ns**	-56.06002197	-16.32681349	-1.466186643
**20 ns**	-69.78089339	-13.74062352	-2.332159866
**40 ns**	-65.45887819	-17.53417521	-2.072311513
**60 ns**	-54.71808866	-12.85776983	-1.931198582
**80 ns**	-58.76995875	-14.35867467	-1.096937619
**100 ns**	-65.53167786	-18.73964582	-1.580307109
**120 ns**	-63.22636945	-22.93473103	-1.211119278
**140 ns**	-65.06175992	-21.59900471	-1.628419506
**160 ns**	-74.30430055	-20.23611992	-0.847748348
**180 ns**	-70.75936902	-18.55209828	-1.080673837
**200 ns**	-52.31722047	-15.35979505	-0.895410655
**Time frame**	**dG_{b}Lipo**	**dG_{b}Packing**	**dG_{b}vdW**
**0 ns**	-21.23819129	-0.548914868	-42.6090217
**20 ns**	-24.23257663	-3.950509813	-45.5633178
**40 ns**	-24.06430969	-4.980824672	-40.269122
**60 ns**	-22.14128499	-5.664209102	-38.4793132
**80 ns**	-24.42943245	-5.159977494	-38.8964222
**100 ns**	-24.86314873	-3.95963453	-46.8788336
**120 ns**	-24.76868254	-1.199727245	-44.7294197
**140 ns**	-24.95749883	-2.117713523	-48.338016
**160 ns**	-26.00665145	-6.614611324	-50.2837019
**180 ns**	-25.25127332	-6.351422375	-47.3747478
**200 ns**	-22.37044373	-2.163565568	-40.1100351

Table S2. Binding free energies of α-Glucosidase_**8g** complex.

Time frame	dG_Bind	Bind_Coulomb	Bind_Hbond
**0 ns**	-74.34001649	-28.98565913	-5.29921623
**20 ns**	-74.81935171	-27.0476851	-3.168594212
**40 ns**	-61.02305249	-21.67519884	-2.551695345
**60 ns**	-52.01293901	-19.462027	-2.199896519
**80 ns**	-51.43814598	-6.931724967	-0.871915741
**100 ns**	-53.45108317	-11.81269687	-1.087630453
**120 ns**	-60.17472947	-12.22312017	-1.374623912
**140 ns**	-74.2585125	-19.5510981	-0.940520194
**160 ns**	-67.11863743	-22.08507845	-1.462782375
**180 ns**	-61.12866433	-13.61230455	-1.106392629
**200 ns**	-69.54123777	-11.10184129	-0.209868385
**Time frame**	**Bind_Lipo**	**Bind_Packing**	**Bind_vdW**
**0 ns**	-31.31270821	-6.335243937	-61.26467282
**20 ns**	-28.74911996	-4.350843786	-47.62001934
**40 ns**	-26.21717862	-6.064331719	-44.18269159
**60 ns**	-19.7644369	-4.480413264	-45.7070177
**80 ns**	-24.34360643	-6.162548566	-40.70543832
**100 ns**	-26.15934106	-3.626822175	-47.3210362
**120 ns**	-29.6896015	-2.560405011	-45.07103614
**140 ns**	-31.77821137	-3.503855469	-55.31829231
**160 ns**	-30.36293505	-1.759423175	-46.3258441
**180 ns**	-26.41553168	-4.711712956	-40.93373536
**200 ns**	-31.17902665	-2.155838615	-45.98826951

General Synthesis Information

General Procedure A for the Synthesis of 3a-3l

In the first step, hydrazinecarbothioamide (**1**, 911.32 mg, 10 mmol, 1 equiv) and various substituted aldehyde (10 mmol, 1 equiv) were combined in fifteen milliliters of methanol, and a few drops of acetic acid were also added. Intermediates (**2a-2l**) were formed when the mixture was refluxed for 4 hours at 60 °C while stirring. Then, using potassium carbonate (2.07 g, 15 mmol, 3 equiv), iodine (7.61 g, 30 mmol, 6 equiv), and 1,4-dioxane as a solvent, the intermediates **2a-2l** (5 mmol, 1 equiv) were cyclized. The reaction mixture was then refluxed for twelve hours at 80 °C. Using TLC, the reaction's progress was monitored, and intermediates (**3a-3l**) were achieved when the reaction was complete. The products were purified using a silica gel column chromatography method with pet ether and ethyl acetate [1].

5-(2-methoxyphenyl)-1,3,4-thiadiazol-2-amine (3a)

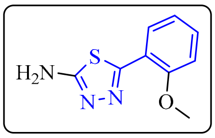

Compound **3a** (yield = 79%) was synthesized according to General Procedure A using (*Z*)-2-(3-methoxybenzylidene)hydrazine-1-carbothioamide (**2a**, 1.05 g, 5 mmol, 1 equiv). **^{1}H NMR (500 MHz, $CDCl_3$) δ** 7.77 (dd, *J* = 8.4, 1.3 Hz, 1H), 7.34 – 7.27 (m, 1H), 7.16 (ddd, *J* = 8.6, 7.5, 1.2 Hz, 1H), 6.96 (dd, *J* = 7.7, 1.2 Hz, 1H), 6.21 (s, 2H), 3.94 (s, 3H). **^{13}C NMR (125 MHz, $CDCl_3$) δ** 170.85, 157.78, 157.07, 131.87, 128.75, 120.87, 120.47, 111.31, 55.76. **HRMS** (ESI) *m/z*: $[M+H]^+$ calcd for $C_9H_{10}N_3OS^+$ 208.0537, found 208.0545.

General Procedure B for the Synthesis of 1-phenyl-3-(pyrimidin-2-yl)-1*H*-pyrazole-4-carbaldehyde (7)

In a round-bottom flask, 1-(pyrimidin-2-yl)ethan-1-one (**4**, 1.22 g, 10 mmol, 1 equiv) and phenylhydrazine (**5**, 1.19 g, 11 mmol, 1.1 equiv) were dissolved in absolute ethanol. Then a few drops of acetic acid were added, and the reaction was refluxed for six hours. After the completion of the reaction, the solution was cooled at room temperature, and the precipitate formed was washed and dried after filtering to afford the intermediate **6**. This intermediate 2-(1-(2-phenylhydrazineylidene)ethyl)pyrimidine (**6**, 1.59 g, 7.5 mmol, 1 equiv) was dissolved in DMF, and then phosphorus oxychloride ($POCl_3$) (2.07 mL, 22.5 mmol, 3 equiv) was added dropwise to the mixture at 0 °C. After addition, this reaction mixture was refluxed for four hours. On the completion of the reaction, the mixture cooled to form a solid crude product, which was then filtered and purified using silica gel chromatography (hexane: ethyl acetate, 7:3) to afford the final product **7** [2].

1-phenyl-3-(pyrimidin-2-yl)-1*H*-pyrazole-4-carbaldehyde (7)

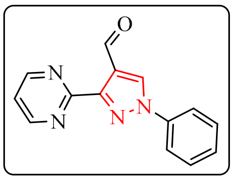

Yield = 85%. **^{1}H NMR (500 MHz, $CDCl_3$) δ** 9.44 (s, 1H), 8.93 (d, *J* = 6.2 Hz, 2H), 8.55 (s, 1H), 7.85 – 7.79 (m, 2H), 7.50 – 7.43 (m, 2H), 7.43 – 7.37 (m, 1H), 7.33 (d, *J* = 8.1 Hz, 1H). **^{13}C NMR (125 MHz, $CDCl_3$) δ** 181.75, 159.61, 158.35, 151.68, 140.87, 134.43, 129.84, 127.70, 121.14, 120.61 (d, *J* = 1.4 Hz). **HRMS** (ESI) *m/z*: $[M+H]^+$ calcd for $C_{14}H_{11}N_4O^+$ 251.0925, found 251.0933.

General Procedure C for the Synthesis of 8a-8l

In the last step, 1-phenyl-3-(pyrimidin-2-yl)-1*H*-pyrazole-4-carbaldehyde (**7**, 750.78 mg, 3 mmol, 1 equiv) was reacted with 5-substituted-1,3,4-thiadiazol-2-amine (**3a-3l**, 3 mmol, 1

equiv) in methanol, and a few drops of glacial acetic acid as a catalyst were also added to the reaction mixture. The reaction mixture was refluxed for seven hours, and the final products (**8a-8l**) were purified using a silica gel column chromatography method with n-hexane and ethyl acetate upon completion of the reaction.

(*Z*)-*N*-(5-(2-methoxyphenyl)-1,3,4-thiadiazol-2-yl)-1-(1-phenyl-3-(pyrimidin-2-yl)-1*H*-pyrazol-4-yl)methanimine (8a)

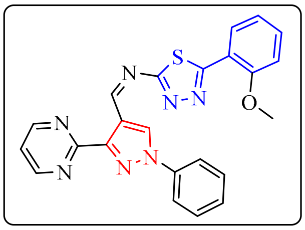

Compound **8a** (yield = 80%) was synthesized according to General Procedure C using 5-(2-methoxyphenyl)-1,3,4-thiadiazol-2-amine (**3a**, 621.75 mg, 3 mmol, 1 equiv). **^{1}H NMR (500 MHz, Chloroform-*d*) δ** 9.37 (s, 1H), 8.92 (d, *J* = 4.0 Hz, 2H), 8.45 (s, 1H), 7.81 (dd, *J* = 8.3, 1.3 Hz, 1H), 7.49 – 7.43 (m, 4H), 7.41 – 7.27 (m, 3H), 7.18 – 7.21 (m, 1H), 7.01 (dd, *J* = 7.7, 1.2 Hz, 1H), 3.94 (s, 3H). **^{13}C NMR (125 MHz, $CDCl_3$) δ** 174.54, 162.15, 160.19, 158.38, 158.16, 156.55, 149.73, 139.73, 133.10, 131.87, 129.84, 129.62, 127.70, 120.95 – 120.52 (m), 120.38, 111.31, 55.76. **HRMS** (ESI) *m/z*: $[M+H]^+$ calcd for $C_{23}H_{18}N_7OS^+$ 440.1276, found 440.1294. Elemental analysis for $C_{23}H_{17}N_7OS$ (%), Calculated: C, 62.86; H, 3.90; N, 22.31; O, 3.64; S, 7.29. Found: C, 62.91; H, 3.95; N, 22.36; O, 3.69; S, 7.34.

(*Z*)-*N*-(5-(3,4-dichlorophenyl)-1,3,4-thiadiazol-2-yl)-1-(1-phenyl-3-(pyrimidin-2-yl)-1*H*-pyrazol-4-yl)methanimine (8b)

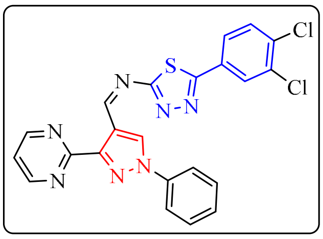

Compound **8b** (yield = 80%) was synthesized according to General Procedure C using 5-(3,4-dichlorophenyl)-1,3,4-thiadiazol-2-amine (**3b**, 738.33 mg, 3 mmol, 1 equiv). **^{1}H NMR (500 MHz, $CDCl_3$) δ** 9.37 (s, 1H), 8.92 (d, *J* = 6.3 Hz, 2H), 8.41 (s, 1H), 8.00 (d, *J* = 2.1 Hz, 1H), 7.85 – 7.78 (m, 3H), 7.72 (d, *J* = 8.2 Hz, 1H), 7.50 – 7.43 (m, 2H), 7.43 – 7.37 (m, 1H), 7.35 – 7.31 (m, 1H). **^{13}C NMR (125 MHz, CDCl3) δ** 174.95, 159.64, 158.91, 152.88 (2C), 149.01, 143.89, 139.03, 135.94, 132.19, 129.83, 128.83, 127.07, 125.97 (2C), 125.91, 121.94, 118.90, 113.70, 111.16, 94.07 (2C). **HRMS** (ESI) *m/z*: $[M+H]^+$ calcd for $C_{22}H_{14}Cl_2N_7S^+$ 478.0408, found 478.0403. Elemental analysis for $C_{22}H_{13}Cl_2N_7S$ (%), Calculated: C, 55.24; H, 2.74; Cl, 14.82; N, 20.50; S, 6.70. Found: C, 55.29; H, 2.79; Cl, 14.87; N, 20.55; S, 6.75; The compound exhibited a retention time of 4.39 min and showed 97.68% chromatographic purity by HPLC-PDA analysis. HPLC-PDA: λ 254 nm, MeOH/MeCN (1:1), Rt: 4.97 min, 97.68%.

(*Z*)-*N*-(5-(4-chlorophenyl)-1,3,4-thiadiazol-2-yl)-1-(1-phenyl-3-(pyrimidin-2-yl)-1*H*-pyrazol-4-yl)methanimine (8c)

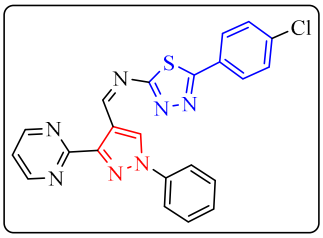

Compound **8c** (yield = 80%) was synthesized according to General Procedure C using 5-(4-chlorophenyl)-1,3,4-thiadiazol-2-amine (**3c**, 635.00 mg, 3 mmol, 1 equiv). **^{1}H NMR (500 MHz, Chloroform-*d*) δ** 9.37 (s, 1H), 8.92 (d, *J* = 4.0 Hz, 2H), 8.64 (s, 1H), 8.28 (dd, *J* = 12.1, 4.6 Hz, 1H), 8.07 (dd, *J* = 12.1, 4.5 Hz, 1H), 7.92 (d, *J* = 8.2 Hz, 1H), 7.89 – 7.85 (m, 2H), 7.75 – 7.70 (m, 2H), 7.64 (d, *J* = 8.2 Hz, 1H), 7.58 (t, *J* = 7.3 Hz, 1H), 7.31 – 7.24 (m, 1H). **^{13}C NMR (125 MHz, CDCl$_3$) δ** 174.70, 164.51, 162.15, 158.38, 156.56, 149.73, 139.73, 136.63, 133.10, 130.66, 129.84, 129.58 (d, *J* = 11.9 Hz), 127.70, 120.81 – 120.52 (m). **HRMS** (ESI) *m/z*: [M+H]$^+$ calcd for $C_{22}H_{15}ClN_7S^+$ 444.0781, found 444.0798. Elemental analysis for $C_{22}H_{14}ClN_7S$ (%), Calculated: C, 59.53; H, 3.18; Cl, 7.99; N, 22.09; S, 7.22. Found: C, 59.58; H, 3.23; Cl, 8.04; N, 22.14; S, 7.27.

(*Z*)-*N*-(5-(2-fluorophenyl)-1,3,4-thiadiazol-2-yl)-1-(1-phenyl-3-(pyrimidin-2-yl)-1*H*-pyrazol-4-yl)methanimine (8d)

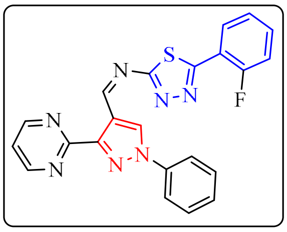

Compound **8d** (yield = 80%) was synthesized according to General Procedure C using 5-(2-fluorophenyl)-1,3,4-thiadiazol-2-amine (**3d**, 585.65 mg, 3 mmol, 1 equiv). **^{1}H NMR (500 MHz, CDCl$_3$) δ** 9.37 (s, 1H), 8.92 (d, *J* = 6.4 Hz, 2H), 8.41 (s, 1H), 7.86 – 7.78 (m, 3H), 7.50 – 7.43 (m, 2H), 7.43 – 7.36 (m, 2H), 7.35 – 7.26 (m, 3H). **^{13}C NMR (125 MHz, CDCl$_3$) δ** 174.72, 163.45, 162.23 – 161.88 (m), 161.46, 158.38, 156.56, 149.73, 139.73, 133.10, 132.78 (d, *J* = 7.2 Hz), 130.10 (d, *J* = 6.0 Hz), 129.84, 127.70, 125.46 (d, *J* = 3.3 Hz), 120.81 – 120.52 (m), 119.86 (d, *J* = 14.1 Hz), 116.22, 116.05. **HRMS** (ESI) *m/z*: [M+H]$^+$ calcd for $C_{22}H_{15}FN_7S^+$ 428.1083, found 428.1094. Elemental analysis for $C_{22}H_{14}FN_7S$ (%), Calculated: C, 61.82; H, 3.30; F, 4.44; N, 22.94; S, 7.50. Found: C, 61.87; H, 3.35; F, 4.49; N, 22.98; S, 7.56.

(*Z*)-4-(5-(((1-phenyl-3-(pyrimidin-2-yl)-1*H*-pyrazol-4-yl)methylene)amino)-1,3,4-thiadiazol-2-yl)benzonitrile (8e)

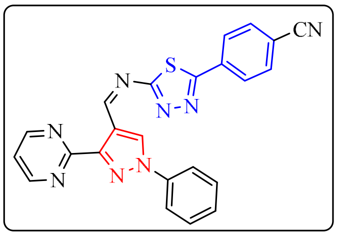

Compound **8e** (yield = 80%) was synthesized according to General Procedure C using 4-(5-amino-1,3,4-thiadiazol-2-yl)benzonitrile (**3e**, 606.70 mg, 3 mmol, 1 equiv). **^{1}H NMR (500 MHz, $CDCl_3$) δ** 9.37 (s, 1H), 8.92 (d, J = 6.3 Hz, 2H), 8.41 (s, 1H), 8.12 – 8.06 (m, 2H), 7.84 – 7.78 (m, 2H), 7.74 – 7.68 (m, 2H), 7.50 – 7.37 (m, 3H), 7.35 – 7.29 (m, 1H). **^{13}C NMR (125 MHz, CDCl3) δ** 168.77, 160.20, 158.81, 156.01, 153.65 (2C), 142.61, 137.79 (3C), 133.61 (2C), 131.34, 130.02, 129.60 (2C), 128.96 (2C), 128.33, 127.28, 121.76, 114.75, 110.54. **HRMS** (ESI) *m/z*: [M+H]$^+$ calcd for $C_{23}H_{15}N_8S^+$ 435.1120, found 435.1140. Elemental analysis for $C_{23}H_{14}N_8S$ (%), Calculated: C, 63.58; H, 3.25; N, 25.79; S, 7.38. Found: C, 63.64; H, 3.28; N, 25.85; S, 7.42.

(*Z*)-5-(5-(((1-phenyl-3-(pyrimidin-2-yl)-1*H*-pyrazol-4-yl)methylene)amino)-1,3,4-thiadiazol-2-yl)benzene-1,2,3-triol (8f)

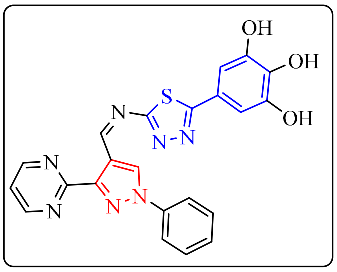

Compound **8f** (yield = 80%) was synthesized according to General Procedure C using 5-(5-amino-1,3,4-thiadiazol-2-yl)benzene-1,2,3-triol (**3f**, 675.67 mg, 3 mmol, 1 equiv). **^{1}H NMR (500 MHz, $CDCl_3$) δ** 9.37 (s, 1H), 8.92 (d, J = 6.3 Hz, 2H), 8.41 (s, 1H), 7.84 – 7.78 (m, 2H), 7.50 – 7.43 (m, 2H), 7.43 – 7.37 (m, 1H), 7.35 – 7.31 (m, 1H), 6.98 (s, 2H), 6.75 (s, 2H), 5.42 (s, 1H). **^{13}C NMR (125 MHz, CDCl3) δ** 168.28, 161.45, 159.00, 157.02 (2C), 151.39 (2C), 147.69, 143.88 (2C), 138.77, 138.70, 133.69, 129.24 (2C), 125.64, 116.44, 92.87 (2C), 91.46, 89.72, 82.63. **HRMS** (ESI) *m/z*: [M+H]$^+$ calcd for $C_{22}H_{16}N_7O_3S^+$ 458.1022, found 458.1035. Elemental analysis for $C_{22}H_{15}N_7O_3S$ (%), Calculated: C, 57.76; H, 3.31; N, 21.43; O, 10.49; S, 7.01. Found: C, 57.83; H, 3.35; N, 21.48; O, 10.53; S, 7.07.

(*Z*)-4-(5-(((1-phenyl-3-(pyrimidin-2-yl)-1*H*-pyrazol-4-yl)methylene)amino)-1,3,4-thiadiazol-2-yl)benzene-1,3-diol (8g)

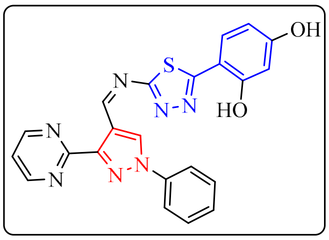

Compound **8g** (yield = 80%) was synthesized according to General Procedure C using 4-(5-amino-1,3,4-thiadiazol-2-yl)benzene-1,3-diol (**3g**, 627.67 mg, 3 mmol, 1 equiv). **^{1}H NMR (500 MHz, Chloroform-*d*) δ** 9.39 (d, J = 17.6 Hz, 2H), 9.00 (s, 1H), 8.92 (d, J = 4.0 Hz, 2H), 8.20 – 8.14 (m, 2H), 8.09 – 8.04 (m, 2H), 7.93 (dd, J = 12.1, 2.2 Hz, 1H), 7.87 (d, J = 8.5 Hz, 1H), 7.60 (dd, J = 8.2, 7.3 Hz, 1H), 7.07 (td, J = 12.1, 1.9 Hz, 1H), 6.60 (dd, J = 8.6, 2.4 Hz, 1H), 6.51 (d, J = 2.4 Hz, 1H). **^{13}C NMR (125 MHz, $CDCl_3$) δ** 174.43, 162.15, 160.57, 160.17, 158.38, 158.19, 156.55, 149.73, 139.73,

133.10, 129.79 (d, J = 12.9 Hz), 127.70, 120.81 – 120.52 (m), 111.34, 108.45, 102.32. **HRMS** (ESI) m/z: $[M+H]^+$ calcd for $C_{22}H_{16}N_7O_2S^+$ 442.1086, found 442.1081. Elemental analysis for $C_{22}H_{15}N_7O_2S$ (%), Calculated: C, 59.86; H, 3.42; N, 22.21; O, 7.25; S, 7.26. Found: C, 59.91; H, 3.46; N, 22.24; O, 7.31; S, 7.31. The compound exhibited a retention time of 5.16 min and showed 97.43% chromatographic purity by HPLC-PDA analysis. HPLC-PDA: λ 254 nm, MeOH/MeCN (1:1), Rt: 5.08 min, 97.43%.

(*Z*)-*N*-(5-(4-methoxyphenyl)-1,3,4-thiadiazol-2-yl)-1-(1-phenyl-3-(pyrimidin-2-yl)-1*H*-pyrazol-4-yl)methanimine (8h)

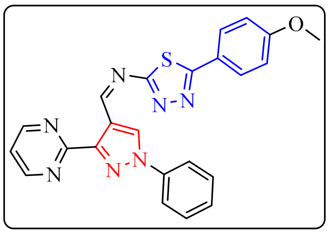

Compound **8h** (yield = 80%) was synthesized according to General Procedure C using 5-(4-methoxyphenyl)-1,3,4-thiadiazol-2-amine (**3h**, 621.75 mg, 3 mmol, 1 equiv). **^{1}H NMR (500 MHz, $CDCl_3$) δ** 9.37 (s, 1H), 8.92 (d, J = 6.7 Hz, 2H), 8.41 (s, 1H), 7.87 – 7.78 (m, 4H), 7.50 – 7.34 (m, 4H), 7.00 – 6.95 (m, 2H), 3.80 (s, 3H). **^{13}C NMR (125 MHz, $CDCl_3$) δ** 174.73, 164.21, 162.13 (d, J = 4.5 Hz), 158.38, 156.56, 149.73, 139.73, 133.10, 129.84, 129.48, 127.70, 125.40, 120.81 – 120.52 (m), 115.00, 55.35. **HRMS** (ESI) m/z: $[M+H]^+$ calcd for $C_{23}H_{18}N_7OS^+$ 440.1287, found 440.1294. Elemental analysis for $C_{23}H_{17}N_7OS$ (%), Calculated: C, 62.86; H, 3.90; N, 22.31; O, 3.64; S, 7.29. Found: C, 62.91; H, 3.95; N, 22.36; O, 3.68; S, 7.32.

(*Z*)-*N*-(5-(4-bromophenyl)-1,3,4-thiadiazol-2-yl)-1-(1-phenyl-3-(pyrimidin-2-yl)-1*H*-pyrazol-4-yl)methanimine (8i)

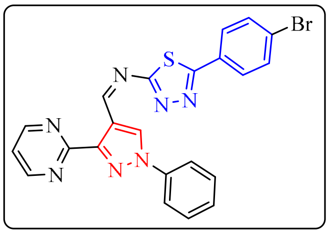

Compound **8i** (yield = 80%) was synthesized according to General Procedure C using 5-(4-bromophenyl)-1,3,4-thiadiazol-2-amine (**3i**, 768.36 mg, 3 mmol, 1 equiv). **^{1}H NMR (500 MHz, $CDCl_3$) δ** 9.37 (s, 1H), 8.92 (d, J = 6.2 Hz, 2H), 8.41 (s, 1H), 7.91 – 7.85 (m, 2H), 7.84 – 7.78 (m, 2H), 7.64 – 7.58 (m, 2H), 7.50 – 7.34 (m, 4H). **^{13}C NMR (125 MHz, $CDCl_3$) δ** 174.70, 164.52, 162.15, 158.38, 156.56, 149.73, 139.73, 133.10, 132.38, 130.88, 130.25, 129.84, 127.70, 126.65, 120.81 – 120.52 (m). **HRMS** (ESI) m/z: $[M+H]^+$ calcd for $C_{22}H_{15}BrN_7S^+$ 488.0279, found 488.0293. Elemental analysis for $C_{22}H_{14}BrN_7S$ (%), Calculated: C, 54.11; H, 2.89; Br, 16.36; N, 20.08; S, 6.56. Found: C, 54.16; H, 2.94; Br, 16.42; N, 20.14; S, 6.61.

(*Z*)-*N*-(5-(3-bromophenyl)-1,3,4-thiadiazol-2-yl)-1-(1-phenyl-3-(pyrimidin-2-yl)-1*H*-pyrazol-4-yl)methanimine (8j)

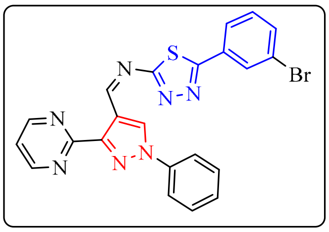

Compound **8j** (yield = 80%) was synthesized according to General Procedure C using 5-(3-bromophenyl)-1,3,4-thiadiazol-2-amine (**3j**, 768.36 mg, 3 mmol, 1 equiv). **^{1}H NMR (500 MHz, Chloroform-*d*) δ** 9.37 (s, 1H), 9.13 (s, 1H), 8.83 (dd, *J* = 5.0, 2.1 Hz, 1H), 8.54 – 8.51 (m, 1H), 8.09 (d, *J* = 6.8 Hz, 1H), 8.01 (dd, *J* = 8.4, 4.9 Hz, 1H), 7.93 – 7.84 (m, 2H), 7.54 – 7.41 (m, 3H), 7.41 – 7.31 (m, 2H), 7.13 – 7.10 (m, 1H). **^{13}C NMR (125 MHz, $CDCl_3$) δ** 174.90, 164.07, 162.15, 158.38, 156.56, 149.73, 139.73, 133.67, 133.10, 132.24 (d, *J* = 18.1 Hz), 130.80, 129.84, 127.70, 126.17, 122.52, 120.81 – 120.52 (m). **HRMS** (ESI) *m/z*: $[M+H]^+$ calcd for $C_{22}H_{15}BrN_7S^+$ 488.0282, found 488.0293. Elemental analysis for $C_{22}H_{14}BrN_7S$ (%), Calculated: C, 54.11; H, 2.89; Br, 16.36; N, 20.08; S, 6.56. Found: C, 54.16; H, 2.94; Br, 16.42; N, 20.14; S, 6.61.

(*Z*)-*N*-(5-(3-fluorophenyl)-1,3,4-thiadiazol-2-yl)-1-(1-phenyl-3-(pyrimidin-2-yl)-1*H*-pyrazol-4-yl)methanimine (8k)

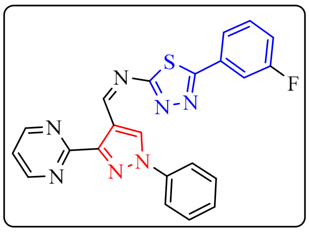

Compound **8k** (yield = 80%) was synthesized according to General Procedure C using 5-(3-fluorophenyl)-1,3,4-thiadiazol-2-amine (**3k**, 585.65 mg, 3 mmol, 1 equiv). **^{1}H NMR (500 MHz, $CDCl_3$) δ** 9.37 (s, 1H), 8.92 (d, *J* = 6.8 Hz, 2H), 8.41 (s, 1H), 7.85 – 7.78 (m, 3H), 7.56 – 7.43 (m, 4H), 7.43 – 7.34 (m, 2H), 7.05 – 7.07 (m, 1H). **^{13}C NMR (125 MHz, CDCL3) δ** 166.46, 163.47 (d, $^1J_{C\text{-}F}$ = 247.50Hz), 160.72, 159.50 (d, $^4J_{C\text{-}F}$ = 1.25 Hz), 152.78, 149.89, 146.83, 138.42, 137.97, 129.89, 129.75 (d, $^3J_{C\text{-}F}$ = 10.0 Hz), 127.64 (d, $^2J_{C\text{-}F}$ = 5.0 Hz), 125.49, 125.31, 120.93, 119.79, 118.28, 114.35 (d, $^2J_{C\text{-}F}$ = 23.75 Hz), 109.05 (d, $^3J_{C\text{-}F}$ = 23.75 Hz), 108.02 (d, $^4J_{C\text{-}F}$ = 3.75 Hz), 107.65, 106.15. **HRMS** (ESI) *m/z*: $[M+H]^+$ calcd for $C_{22}H_{15}FN_7S^+$ 428.1094, found 428.1088. Elemental analysis for $C_{22}H_{14}FN_7S$ (%), Calculated: C, 61.82; H, 3.30; F, 4.44; N, 22.94; S, 7.50. Found: C, 61.87; H, 3.36; F, 4.49; N, 22.98; S, 7.55. The compound exhibited a retention time of 4.53 min and showed 98.13% chromatographic purity by HPLC-PDA analysis. HPLC-PDA: λ 254 nm, MeOH/MeCN (1:1), Rt: 3.89 min, 98.13%.

(*Z*)-*N*,*N*-dimethyl-4-(5-(((1-phenyl-3-(pyrimidin-2-yl)-1*H*-pyrazol-4-yl)methylene)amino)-1,3,4-thiadiazol-2-yl)aniline (8l)

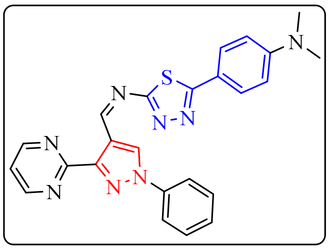

Compound **8l** (yield = 80%) was synthesized according to General Procedure C using 5-(4-(dimethylamino)phenyl)-1,3,4-thiadiazol-2-amine (**3l**, 660.88 mg, 3 mmol, 1 equiv). **^{1}H NMR (500 MHz, Chloroform-*d*) δ** 9.37 (s, 1H), 8.92 (d, *J* = 4.0 Hz, 2H), 8.41 (s, 1H), 8.23 (dd, *J* = 12.1, 4.5 Hz, 1H), 7.92 (dd, *J* = 12.1, 4.5 Hz, 1H), 7.86 – 7.78 (m, 3H), 7.49 – 7.45 (m, 3H), 7.43 – 7.37 (m, 1H), 7.09 (d, *J* = 7.6 Hz, 1H), 2.83 (s, 6H). **^{13}C NMR (125 MHz, $CDCl_3$) δ** 174.70, 164.33, 162.15, 158.38, 156.56, 152.20, 149.73, 139.73, 133.10, 129.84, 128.16, 127.70, 124.01, 120.81 – 120.52 (m), 113.93, 40.31. **HRMS** (ESI) *m/z*: $[M+H]^+$ calcd for $C_{24}H_{21}N_8S^+$ 453.1603, found 453.1610. Elemental analysis for $C_{24}H_{20}N_8S$ (%), Calculated: C, 63.70; H, 4.45; N, 24.76; S, 7.08. Found: C, 63.75; H, 4.49; N, 24.79; S, 7.14.

Table S3: ADMET analysis of synthesized compounds

**Physicochemical Properties**					
**Compound**	**8g**	**8k**	**8b**	**8e**	**Acarbose**
**Molecular Weight (g/mol)**	441.47	427.46	478.36	438.48	645.60
**Number of Heavy Atoms**	32	31	32	32	44
**Number of Aromatic Heavy Atoms**	28	28	28	28	0
**Fraction Csp3**	0.00	0.00	0.00	0.00	0.92
**Number of Rotatable Bonds**	5	5	5	5	9
**Number of Hydrogen Bond Acceptors**	8	7	6	7	19
**Number of Hydrogen Bond Donors**	2	0	0	0	14
**Molar Refractivity**	121.33	117.15	127.21	121.90	136.69
**TPSA (Å²)**	150.44	109.98	109.98	133.77	321.17

Pharmacokinetics					
**GI Absorption**	Low	High	Low	Low	Low
**BBB Permeant**	No	No	No	No	No
**P-gp Substrate**	Yes	Yes	No	Yes	Yes
**CYP1A2 Inhibitor**	No	No	No	No	No
**CYP2C19 Inhibitor**	No	No	No	No	No
**CYP2C9 Inhibitor**	Yes	Yes	Yes	Yes	No
**CYP2D6 Inhibitor**	No	No	No	No	No
**CYP3A4 Inhibitor**	No	No	No	Yes	No
**Drug likeness**					
**Lipinski**	No	Yes	Yes	Yes	No
**Ghose**	Yes	Yes	No	Yes	No
**Veber**	No	Yes	Yes	Yes	No
**Egan**	No	Yes	No	No	No
**Muegge**	No	Yes	Yes	Yes	No
**Bioavailability Score**	0.55	0.55	0.55	0.55	0.17
**Medicinal chemistry**					
**PAINS Alert**	0 alert	0 alert	0 alert	0 alert	0 alert
**Brenk Alert**	1 alert	1 alert	1 alert	1alert	1 alert
**Lead likeness**	No	No	No	No	No
**Synthetic Accessibility**	3.69	3.54	3.59	3.65	0.17

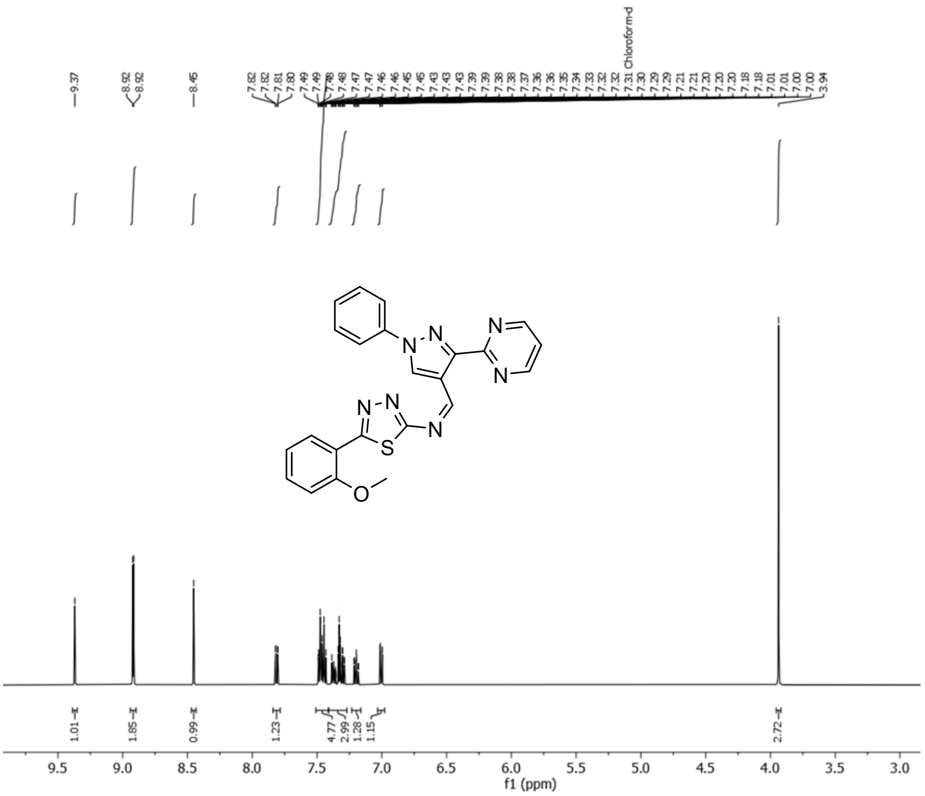

Figure S2. The 1H NMR for compound (Z)-N-(5-(2-methoxyphenyl)-1,3,4-thiadiazol-2-yl)-1-(1-phenyl-3-(pyrimidin-2-yl)-1H-pyrazol-4-yl)methanamine (**8a**)

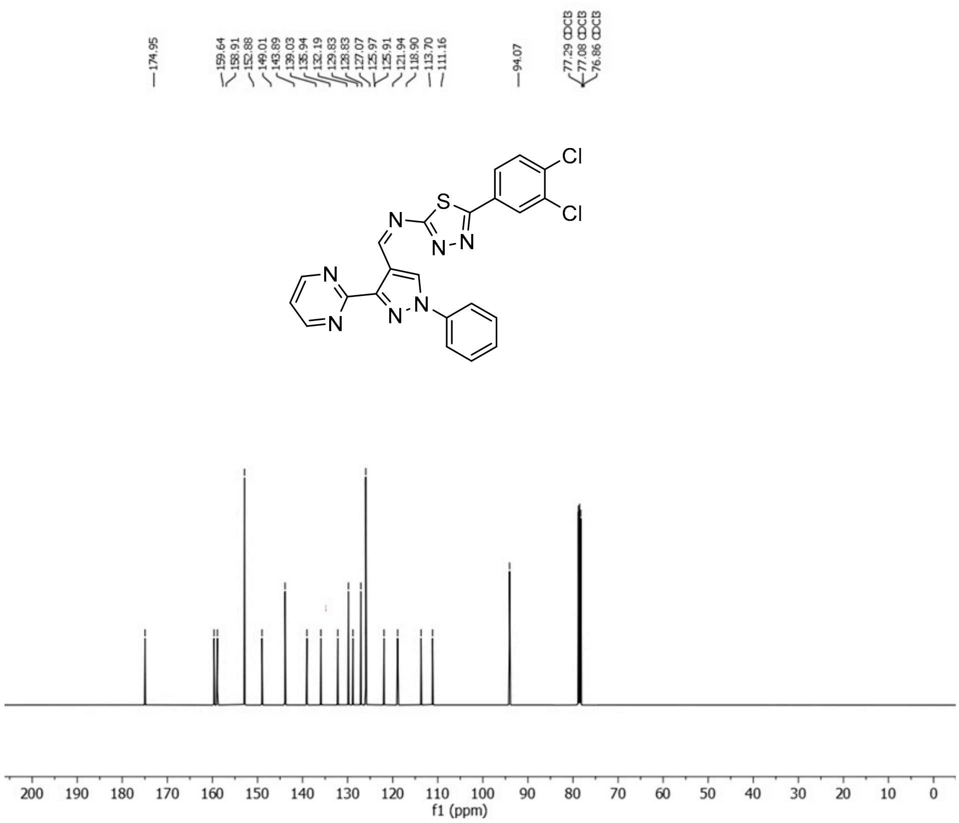

Figure S3. The 13C NMR for compound (Z)-N-(5-(3,4-dichlorophenyl)-1,3,4-thiadiazol-2-yl)-1-(1-phenyl-3-(pyrimidin-2-yl)-1H-pyrazol-4-yl)methanimine (**8b**)

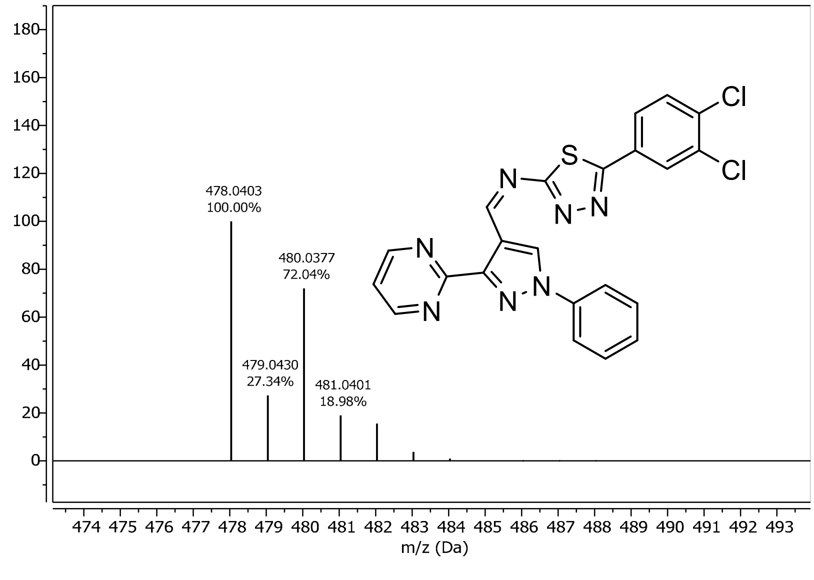

Figure S4. The HRMS for compound (Z)-N-(5-(3,4-dichlorophenyl)-1,3,4-thiadiazol-2-yl)-1-(1-phenyl-3-(pyrimidin-2-yl)-1H-pyrazol-4-yl)methanimine (**8b**)

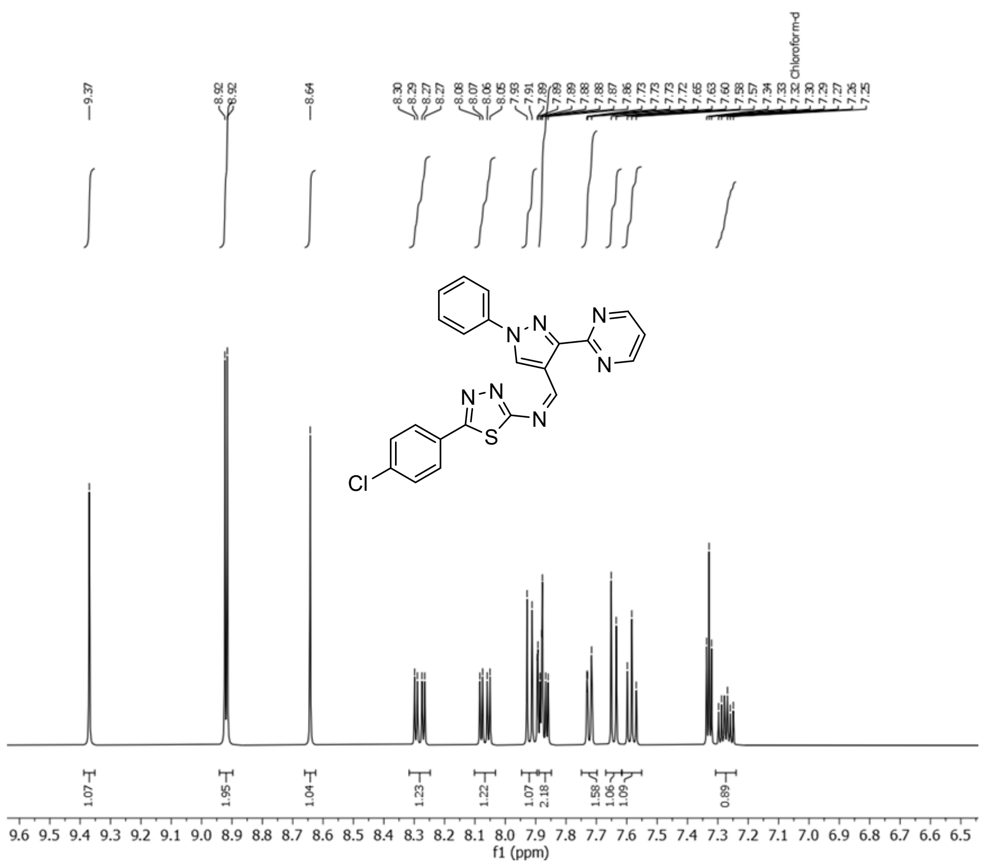

Figure S5. The 1H NMR for compound (Z)-N-(5-(4-chlorophenyl)-1,3,4-thiadiazol-2-yl)-1-(1-phenyl-3-(pyrimidin-2-yl)-1H-pyrazol-4-yl)methanimine (**8c**)

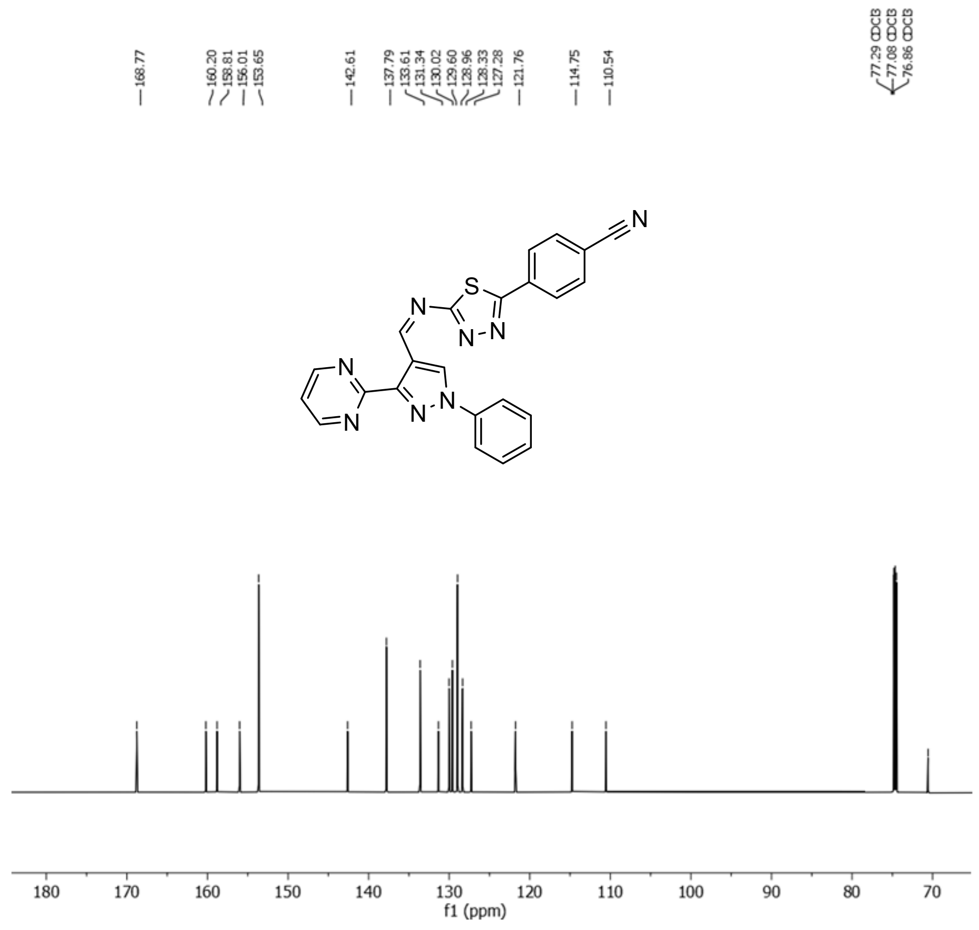

Figure S6. The 13C NMR for compound (Z)-4-(5-(((1-phenyl-3-(pyrimidin-2-yl)-1H-pyrazol-4-yl)methylene)amino)-1,3,4-thiadiazol-2-yl)benzonitrile (**8e**)

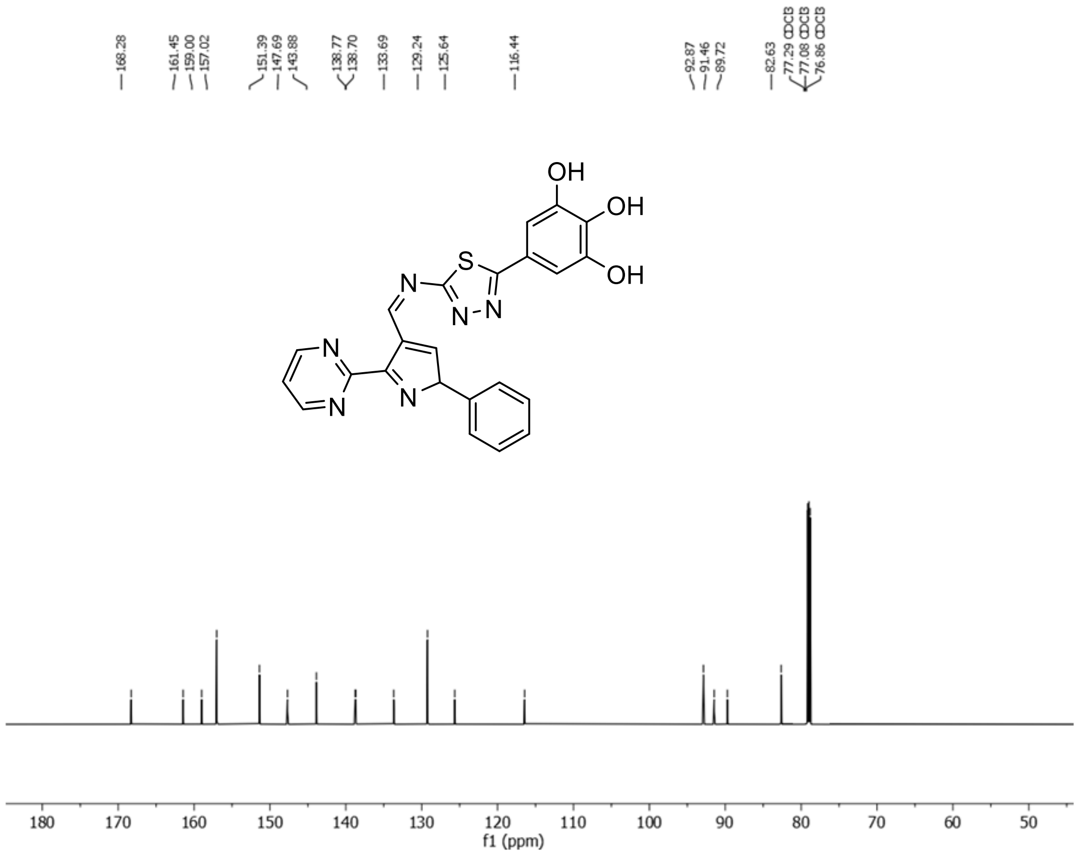

Figure S7. The 13C NMR for compound (Z)-5-(5-(((2-phenyl-5-(pyrimidin-2-yl)-2H-pyrrol-4-yl)methylene)amino)-1,3,4-thiadiazol-2-yl)benzene-1,2,3-triol (**8f**)

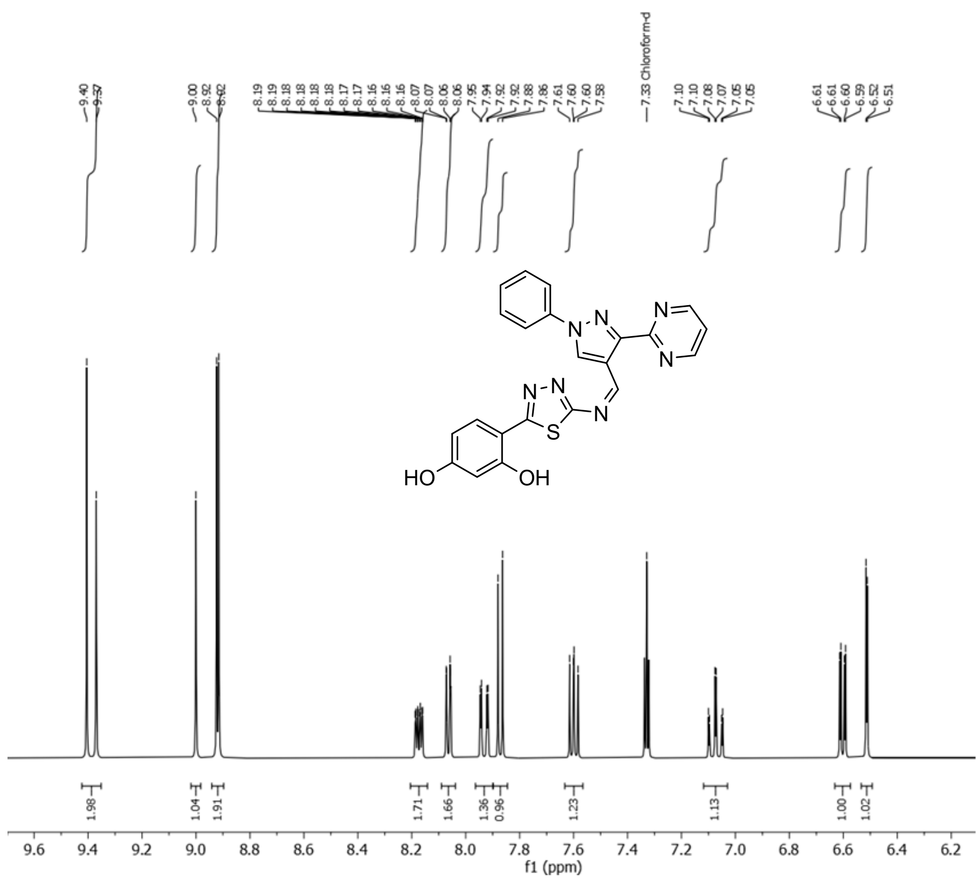

Figure S8. The 1H NMR for compound (Z)-4-(5-(((1-phenyl-3-(pyrimidin-2-yl)-1H-pyrazol-4-yl)methylene)amino)-1,3,4-thiadiazol-2-yl)benzene-1,3-diol (**8g**)

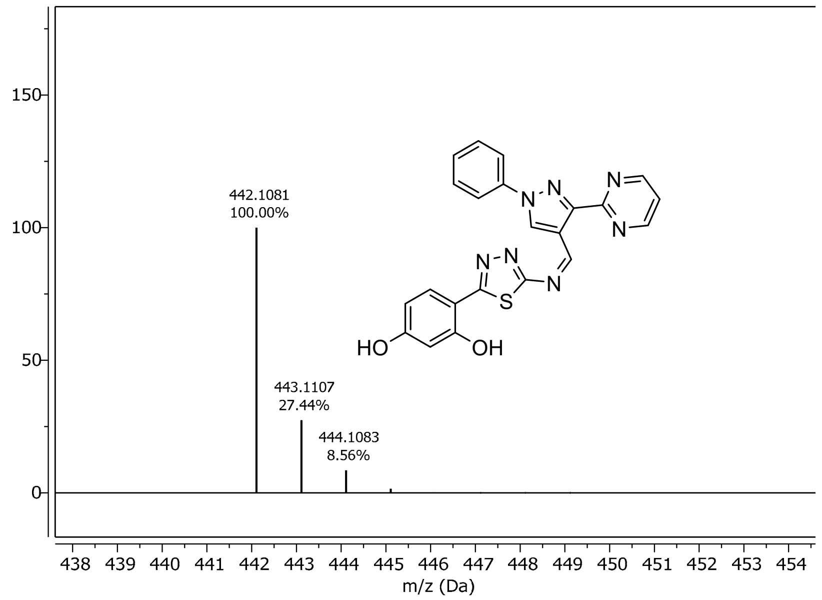

Figure S9. The HRMS for compound (Z)-4-(5-(((1-phenyl-3-(pyrimidin-2-yl)-1H-pyrazol-4-yl)methylene)amino)-1,3,4-thiadiazol-2-yl)benzene-1,3-diol (**8g**)

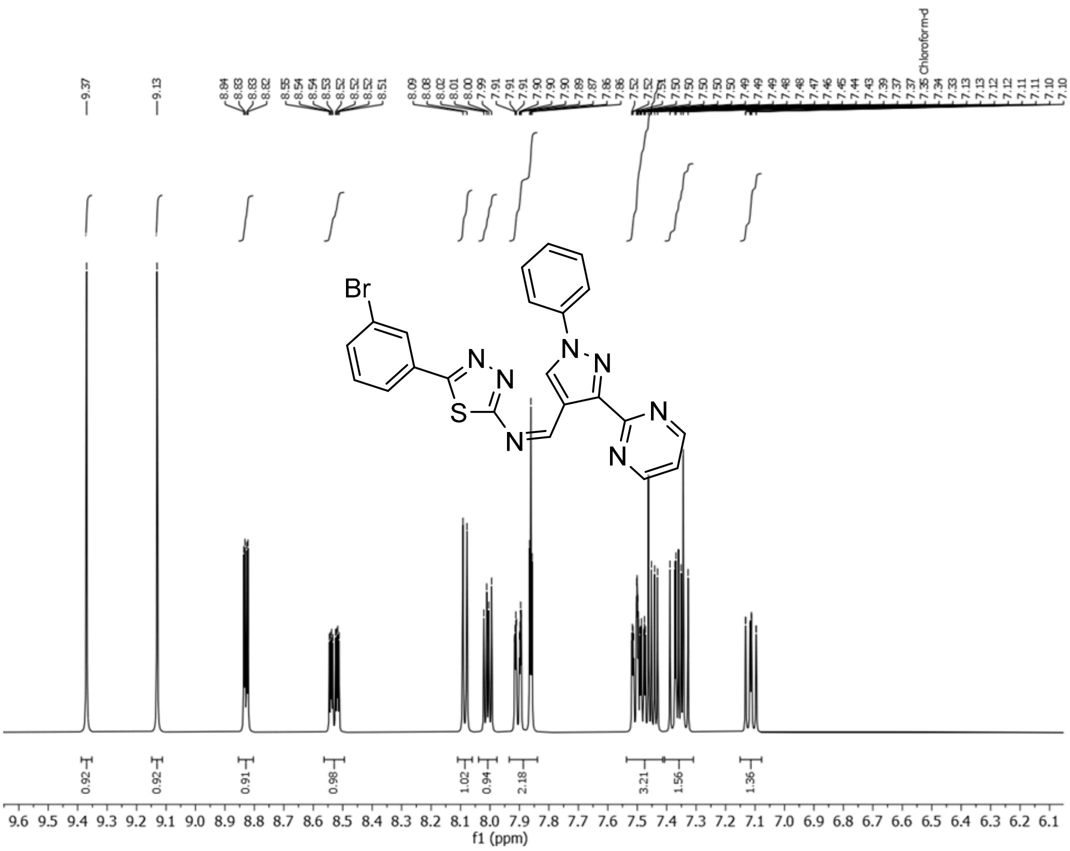

Figure S10. The 1H NMR for compound (Z)-N-(5-(3-bromophenyl)-1,3,4-thiadiazol-2-yl)-1-(1-phenyl-3-(pyrimidin-2-yl)-1H-pyrazol-4-yl)methanimine (**8j**)

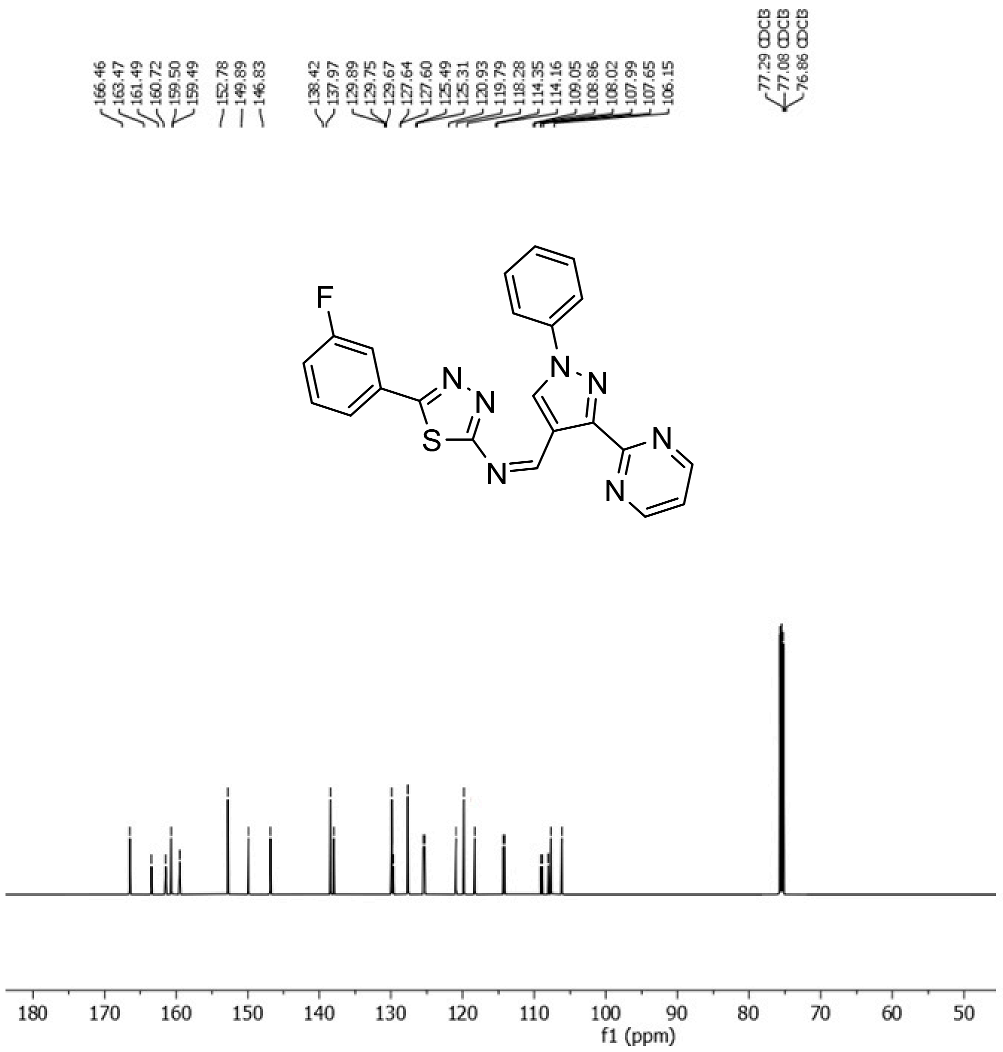

Figure S11. The 13C NMR for compound (Z)-N-(5-(3-fluorophenyl)-1,3,4-thiadiazol-2-yl)-1-(1-phenyl-3-(pyrimidin-2-yl)-1H-pyrazol-4-yl)methanimine (**8k**)

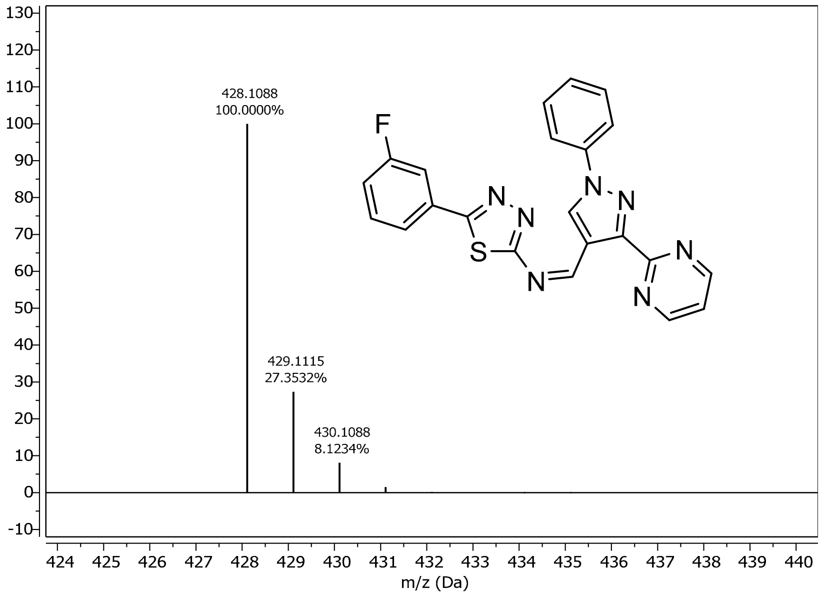

Figure S12. The HRMS for compound (Z)-N-(5-(3-fluorophenyl)-1,3,4-thiadiazol-2-yl)-1-(1-phenyl-3-(pyrimidin-2-yl)-1H-pyrazol-4-yl)methanimine (**8k**)

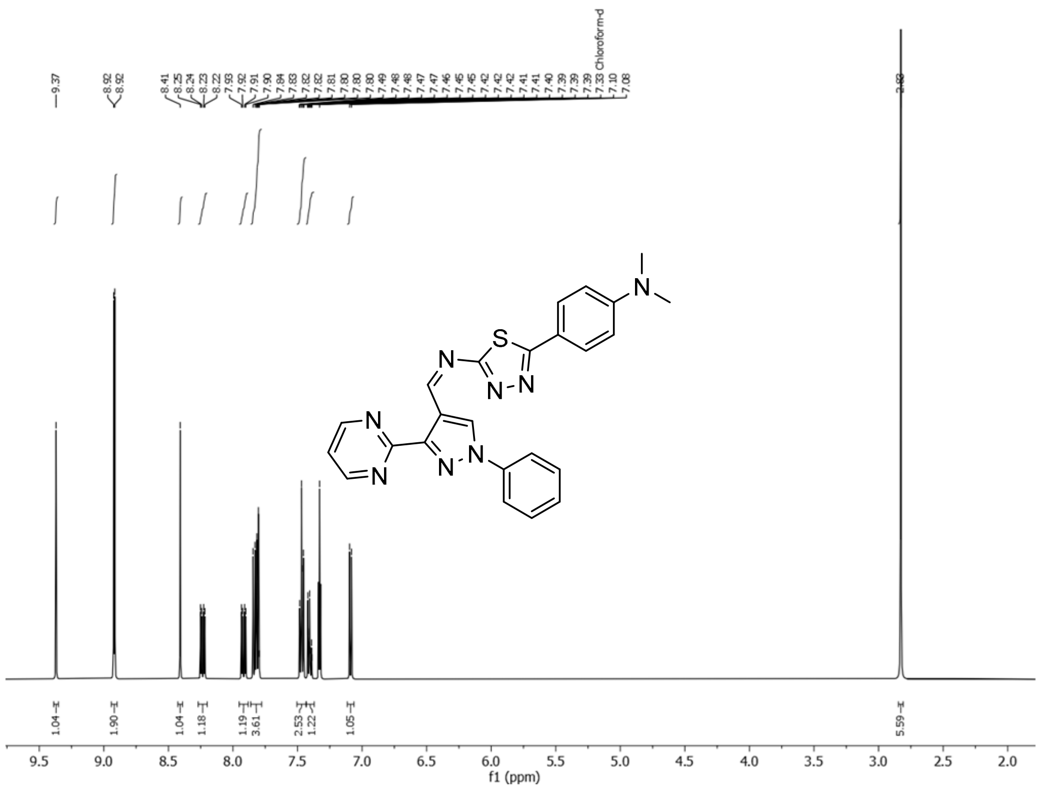

Figure S13. The 1H NMR for compound (Z)-N,N-dimethyl-4-(5-(((1-phenyl-3-(pyrimidin-2-yl)-1H-pyrazol-4-yl)methylene)amino)-1,3,4-thiadiazol-2-yl)aniline (**8l**)

References:

[1] Abbasi, S. A., Rahim, F., Hussain, R., Khan, S., Ullah, H., Iqbal, T., ... & Ansari, M. J. (2024). Synthesis of modified 1, 3, 4-thiadiazole incorporating substituted thiosemicarbazide derivatives: Elucidating the in vitro and in silico studies to develop promising anti-diabetic agent. *Results in Chemistry*, *8*, 101556.

[2] Konkala, V. S., & Dubey, P. K. (2017). Urea/thiourea: efficient, inexpensive and reusable catalysts for the synthesis of pyrazole derivatives with 2-iminothiazolidin-4-one and 2, 4-thiazolidinediones under solvent-free conditions. *Molecular Diversity*, *21*(2), 283-291.
